# Whole set of constitutive promoters for RpoN sigma factor and the regulatory role of its enhancer protein NtrC in *Escherichia coli* K-12

**DOI:** 10.1099/mgen.0.000653

**Published:** 2021-11-17

**Authors:** Tomohiro Shimada, Shun Furuhata, Akira Ishihama

**Affiliations:** ^1^​ School of Agriculture, Meiji University, Kawasaki, Kanagawa, Japan; ^2^​ Micro-Nanotechnology Research Center, Hosei University, Koganei, Tokyo, Japan

**Keywords:** *Escherichia coli*, gSELEX, nitrogen metabolism, NtrC, RNA polymerase, RpoN sigma factor

## Abstract

The promoter selectivity of *Escherichia coli* RNA polymerase (RNAP) is determined by its promoter-recognition sigma subunit. The model prokaryote *E. coli* K-12 contains seven species of the sigma subunit, each recognizing a specific set of promoters. Using genomic SELEX (gSELEX) screening *in vitro*, we identified the whole set of ‘constitutive’ promoters recognized by the reconstituted RNAP holoenzyme alone, containing RpoD (σ^70^), RpoS (σ^38^), RpoH (σ^32^), RpoF (σ^28^) or RpoE (σ^24^), in the absence of other supporting regulatory factors. In contrast, RpoN sigma (σ^54^), involved in expression of nitrogen-related genes and also other cellular functions, requires an enhancer (or activator) protein, such as NtrC, for transcription initiation. In this study, a series of gSELEX screenings were performed to search for promoters recognized by the RpoN RNAP holoenzyme in the presence and absence of the major nitrogen response enhancer NtrC, the best-characterized enhancer. Based on the RpoN holoenzyme-binding sites, a total of 44 to 61 putative promoters were identified, which were recognized by the RpoN holoenzyme alone. In the presence of the enhancer NtrC, the recognition target increased to 61–81 promoters. Consensus sequences of promoters recognized by RpoN holoenzyme in the absence and presence of NtrC were determined. The promoter activity of a set of NtrC-dependent and -independent RpoN promoters was verified *in vivo* under nitrogen starvation, in the presence and absence of RpoN and/or NtrC. The promoter activity of some RpoN-recognized promoters increased in the absence of RpoN or NtrC, supporting the concept that the promoter-bound NtrC-enhanced RpoN holoenzyme functions as a repressor against RpoD holoenzyme. Based on our findings, we propose a model in which the RpoN holoenzyme fulfils the dual role of repressor and transcriptase for the same set of genes. We also propose that the promoter recognized by RpoN holoenzyme in the absence of enhancers is the ‘repressive’ promoter. The presence of high-level RpoN sigma in growing *E. coli* K-12 in rich medium may be related to the repression role of a set of genes needed for the utilization of ammonia as a nitrogen source in poor media. The list of newly identified regulatory targets of RpoN provides insight into *E. coli* survival under nitrogen-depleted conditions in nature.

## Data Summary

The genomic SELEX data for RpoN RNA polymerase (RNAP) holoenzyme (https://shigen.nig.ac.jp/ecoli/tec/download/export/1/RpoN), NtrC (https://shigen.nig.ac.jp/ecoli/tec/download/export/2/NtrC) and RpoN RNAP holoenzyme+NtrC (https://shigen.nig.ac.jp/ecoli/tec/download/export/1/RpoNNtrC) have been deposited in the Transcription Factor Profiling of *Escherichia coli* (TEC) database at the National Institute of Genetics, Japan (https://shigen.nig.ac.jp/ecoli/tec/).

**Impact Statement**

The promoter selectivity of *Escherichia coli* RNA polymerase (RNAP) is determined by the promoter-recognition sigma subunit. RpoN sigma (σ^54^), involved in the expression of nitrogen-related genes and also other cellular functions, requires an enhancer, such as NtrC, for transcription initiation. Using the genomic-SELEX-chip method, we identified the number of target genes or operons as 44–61, 30–41 and 61–81 for RpoN RNAP holoenzyme, NtrC and RpoN RNAP holoenzyme+NtrC, respectively. Newly identified NtrC-dependent RpoN target genes include not only nitrogen-related genes but also nitrogen-related metabolism genes, such as those involved in carbon source metabolism. Based on our findings, we propose a dual function for the RNAP RpoN holoenzyme, as a repressor (in the absence of NtrC) and as a NtrC-activated transcriptase. This type of promoter, recognized by the RpoN holoenzyme alone, was termed as a repressive promoter.

## Introduction

The specificity of the promoter recognition of RNA polymerase (RNAP), responsible for environmental changes in bacteria, is modulated by replacement of the σ subunit, which controls differential gene expression [1, 2]. The sigma subunit σ^54^_,_ encoded by the *rpoN* gene, was first discovered during an analysis of glutamine synthetase and nitrogen assimilation in enteric bacteria [3]. Subsequent studies have confirmed its role in nitrogen assimilation but have also shown that it is involved in a variety of nitrogen-related, and seemingly unrelated, functions [4–6].

Ammonia is considered the preferred nitrogen source for *Escherichia coli* grown in minimal medium [7]. Under nitrogen depletion, one of the enhancers of NtrC encoded by *glnG* is activated by the phosphorylation of the kinase NtrB encoded by *glnL* [8]. These two proteins form the NtrBC two-component system (TCS). Phosphorylated NtrC binds to the nitrogen-regulated gene promoters, and in conjunction with the RpoN holoenzyme, transforms the promoter closed complex to an open complex [8–11]. NtrC directly or indirectly controls the majority of nitrogen-regulated genes [6, 12]. Based on this well-characterized NtrC-dependent transcription initiation system as a model, the RpoN RNAP holoenzyme is believed to be unique, with respect to the requirement of the enhancer (or activator), as follows: RNAP RpoN holoenzyme (RpoN-core enzyme complex) binds to the promoter to form an inactive closed complex, which is converted into the active open promoter complex after addition of hydrolysing ATP, with the help of a distinct class of transcriptional activators called enhancer-binding proteins [13, 14]. Moreover, the requirement of enhancers is unique to RpoN and not the other six sigma factors [15]. At present, 12 transcription factors (TFs) are known to be *E. coli* enhancers, belonging to two families: AtoC, NorR, NtrC, PrpR, PspF, QseF, RtcR and ZraR, which belong to the NtrC family, and DhaR, FhlA, HyfR and TyrR, which belong to the TyrR family [16].

To understand the regulatory role of RpoN *in vivo*, attempts have been made to identify RpoN-regulated promoters, such as with ChIP-seq analysis for detecting the RpoN-binding sites on the *E. coli* genome [17] and RNA-seq analysis to determine the mRNA levels after deletion of RpoN [18]. These genome-wide approaches have indicated the presence of novel RpoN targets. However, it is difficult to distinguish between the direct and indirect effects of the RpoN holoenzyme and/or the enhancer. For instance, the intracellular concentrations of RNAP sigma factor and TFs change depending on the growth phase and growth conditions [19, 20]. Therefore, it is difficult to identify the whole set of direct regulatory targets of NtrC *in vivo*, even though the genome-wide transcriptome [21] and genome-wide distribution [22] have been analysed. To avoid the problems associated with *in vivo* experiments, we performed genomic SELEX (gSELEX) screening of genomic DNA sequences recognized by the RNAP holoenzyme containing RpoN sigma (without other sigma factors) in the presence or absence of a single species NtrC enhancer. The original SELEX screening uses synthetic oligonucleotides with all possible sequences, and is able to identify the target DNA sequence. However, after a computer-based homology search for the consensus sequences, it is difficult to identify the whole set of target genes from the entire genome, because of difficulty in distinguishing positive target and false positive; whereas the gSELEX screening system uses genome fragments with all possible target sequences. The gSELEX screening system was developed to directly identify DNA sequences recognized *in vitro* by DNA-binding TFs [23, 24] and successfully applied to identify regulatory targets of more than 200 TFs from a single species: *E. coli* K-12 W3350 [16, 25]. Using this gSELEX method, we also identified the whole set of promoters recognized by a single species of sigma factor, including the major sigma subunit RpoD [26] and four species of the minor sigma subunits, RpoS, RpoH, RpoF and RpoE [27]. Promoter search using the gSELEX system enabled the detection of the whole set of constitutive promoters recognized by each RNAP holoenzyme alone in the absence of other supporting factors, as well as in the absence of interfering proteins, including other sigma factors. Thus, the numbers of constitutive promoters for each sigma factor identified were as follows: 1320 for RpoD, 235 for RpoS, 331 for RpoH, 260 for RpoF and 493 for RpoE [26–28]. Based on the list of constitutive promoters, we could also predict the ‘inducible’ promoters recognized and activated in the presence of additional supporting factors. Under *in vivo* conditions, it is impossible to obtain the whole set of binding sites for both RNAP and TFs. In addition, the transcription-related data listed in the databases include different levels of accuracy. For instance, a number of TF-binding sites are estimated *in silico*, relying on consensus sequences that often include inaccurate predictions. Another significant problem originates from the use of various *E. coli* strains with different genetic backgrounds and the use of different culture conditions in each experiment.

In this study, we identified the whole set of RpoN-dependent promoters and the whole set of NtrC-binding sites using gSELEX screening. Furthermore, gSELEX analysis of the RpoN holoenzyme was performed in the presence of NtrC to identify the RpoN promoters regulated by the NtrC enhancer. The promoter activity of some RpoN promoters was examined using a gel-shift assay *in vitro* and reverse-transcription quantitative real-time PCR (RT-qPCR) assay *in vivo*. The promoter activity of some of the promoters recognized by the RpoN holoenzyme alone increased in the absence of RpoN. Furthermore, the binding of the RpoD holoenzyme to the test promoter was interfered with by the binding of the NtrC-enhanced RpoN holoenzyme to the promoter, suggesting a repressor function of the RpoN holoenzyme with competition against other RNAP holoenzymes. We designated this promoter as a repressive promoter, alongside the constitutive promoter for RpoN-family sigma factors. The whole set of repressive promoters described herein provides fundamental catalogues for the promoters recognized by RpoN sigma factors and a useful resource for further analysis combined with other enhancers.

## Methods

### Bacterial strains and plasmids

*E. coli* K-12 W3350 type-A, containing the full set of seven sigma factors [29], was used for the purification of RNAP and as a template DNA for the gSELEX screening of RpoN promoters and NtrC target genes. *E. coli* DH5α was used for plasmid amplification. *E. coli* BL21(DE3) was used for the expression and purification of sigma N and sigma D, core enzyme subunit proteins, and NtrC. Expression plasmids for the core enzyme subunits and sigma N subunits (pRpoA, pRpoB, pRpoC, pRpoD and pRpoN) and NtrC (pNtrC) were constructed by ligating the corresponding coding sequences, which were prepared via PCR amplification of the *E. coli* K-12 W3350 type-A genomic DNA as a template, into the pET21 expression vector, according to a standard procedure used for the expression of sigma and TFs [30, 31]. *E. coli* BW25113 [32] and its single-gene knockout mutants, JW3169 for *rpoN* and JW3839 for *ntrC* [33], were obtained from the *E. coli* Stock Centre (National Bio-Resource Centre, Japan).

Cells were grown in LB medium or 3 mM NH_4_Cl (for nitrogen-starvation experiments) Gutnick minimal medium [34] (33.8 mM KH_2_PO_4_, 77.5 mM K_2_HPO_4_, 5.74 mM K_2_SO_4_ and 0.41 mM MgSO_4_ supplemented with Ho-LE trace elements and 0.2%, w/v, glucose), using NH_4_Cl as the sole nitrogen source, at 37 °C with constant shaking at 150 r.p.m. When necessary, 20 µg kanamycin ml^−1^ was added to the medium. Cell growth was monitored by measuring the turbidity at 600 nm.

### Purification of core RNAP

RNAP was purified from log-phase cells of *E. coli* K-12 W3350 using a standard procedure [35]. The native core was separated from the holoenzymes by passing the purified RNAP through a P11-phosphocellulose column in the presence of 50% (v/v) glycerol. To remove trace amounts of the core-enzyme-associated sigma factors, the purified RNAP in the storage buffer containing 50% (v/v) glycerol was dialysed against the same buffer containing 5% (v/v) glycerol and fractionated by P11-phosphocellulose column chromatography in the presence of 5% (v/v) glycerol. The level of remaining sigma factors was less than 0.1 %, if any, as verified using SDS-PAGE gels by both protein staining with a silver reagent and immunostaining with antibodies against each of the seven sigma factors.

### Purification of core and sigma subunits

The core enzyme subunits (RpoA, RpoB, RpoC and RpoZ) were expressed using corresponding expression plasmids and purified by two cycles of column chromatography using DEAE (DE52) and P11-phosphocellulose [35]. The sigma subunits were expressed and purified via ion-exchange column chromatography using DE52 and P11, followed by a Sephacryl S-300 gel filtration column. The purified sigma and core subunit proteins were over 99% pure, as determined by both protein staining and immunostaining of SDS-PAGE gels.

### Purification of antibodies

Antibodies against core enzyme subunits were produced in rabbits by injecting purified proteins [36, 37]. Antibodies against each RNAP protein were produced in two rabbits, and after the examination of antibody activity using immunoblot analysis, a batch with higher activity was used in this study. The anti-RpoD, anti-RpoS, anti-RpoN, anti-RpoH, anti-RpoF, anti-RpoE, anti-FecI and anti-RpoC used in this study did not cross-react with each other. These antibodies were produced by the Nippon Institute for Biological Science and the Animal Laboratory of Mitsubishi Chemical Medience.

### gSELEX screening of the binding sequences of RpoN RNAP holoenzyme and NtrC

gSELEX screening was performed using a standard procedure [23, 24]. A mixture of DNA fragments of the *E. coli* K-12 W3350 genome was prepared by sonicating purified genomic DNA and cloning it into multi-copy plasmid pBR322 at the *Eco*RV site. In each gSELEX screening, the DNA mixture was regenerated by PCR using a pair of primers with the flanking sequences of pBR322 *Eco*RV. For gSELEX screening, 5 pmol of the mixture of the DNA fragments and 10 pmol RpoN RNAP holoenzyme or NtrC were mixed in a binding buffer (10 mM Tris-HCl, pH 7.8 at 4 °C, 3 mM magnesium acetate, 150 mM NaCl and 1.25 mg BSA ml^−1^) and incubated for 30 min at 37 °C. For reconstitution of the RpoN holoenzyme, the sigma-free core enzyme and fourfold molar excess of RpoN sigma subunit were mixed and incubated. For NtrC, acetylphosphate (0.1 mM) was added for NtrC auto-phosphorylation. The DNA-RpoN RNAP mixture was treated with anti-RpoC antibody and A/G beads, whereas the DNA-NtrC mixture was treated with a Ni-nitrilotriacetic acid (NTA) agarose column for purified DNA–test protein complexes. DNA fragments recovered from the complexes were PCR amplified and subjected to the next cycle of gSELEX to enrich the test protein-bound DNA fragments.

For gSELEX-chip analysis, DNA samples were isolated from the DNA-protein complexes at the final state of gSELEX, PCR-amplified and labelled with Cy5, while the original DNA library was labelled with Cy3. The fluorescenty labelled DNA mixtures were hybridized to a DNA microarray consisting of 43 450 species of 60 bp long DNA probes, which were designed to cover the entire *E. coli* K-12 MG1655 genome at 105 bp intervals (Agilent). The fluorescence intensity of the test sample at each probe was normalized to that of the corresponding peak of the original library. After the normalization of each pattern, the Cy5/Cy3 ratio was measured and plotted along the *E. coli* K-12 MG1655 genome. The gene organization was almost identical between the two well-characterized *E. coli* K-12 strains, except for a long-range inversion between the *rrnD* and *rrnE* operons.

### Consensus-sequence analysis

To analyse the RpoN promoter motif and the NtrC binding sequence, each set of 500 bp binding sequences centred on the binding peak identified by gSELEX-chip was analysed using the program meme suite [38]. The sequences were aligned, and a consensus-sequence logo was created using weblogo (http://weblogo.berkeley.edu/logo.cgi).

### Gel-shift assay

The gel-shift assay was performed according to standard procedures [39]. Probes of the NtrC-dependent RpoN holoenzyme-binding target sequences were generated by PCR amplification using a pair of primers (Table S1a, available with the online version of this article) and *Ex Taq* DNA polymerase (TaKaRa). A mixture of each probe and RpoN holoenzyme, NtrC and RpoD holoenzyme was incubated at 37 °C for 30 min in the gel-shift buffer with 25 mM acetylphosphate and ATP. After the addition of the DNA loading solution, the mixture was directly subjected to 3.5% PAGE. DNA in gels was stained with GelRed (Biotium) and detected using LuminoGraph I (Atto).

### RT-qPCR analysis

RT-qPCR analysis was performed according to a standard procedure [40]. The nitrogen replete and deplete conditions were made according to Brown *et al.* with some modifications [22]. *E. coli* cells were inoculated in Gutnick minimal medium supplemented with 0.2% glucose and 3 mM NH_4_Cl at 37 °C under aeration with constant shaking at 150 r.p.m. until an OD_600_ of 0.4 (nitrogen replete condition in exponential phase) or 0.9 (nitrogen deplete condition, 20 min after growth stopped) was reached. Then, the total RNA was extracted. Total RNA was transcribed to cDNA with random primers using the THUNDERBIRD SYBR qPCR RT kit (Toyobo). qPCR (quantitative real-time PCR) was conducted using THUNDERBIRD SYBR qPCR mix (Toyobo) and was performed using the LightCycler 96 system (Roche). The primer pairs used are listed in Table S1(b). The cDNA templates were serially diluted fourfold and used for qPCR assays. The qPCR mixtures, each containing 10 µl THUNDERBIRD SYBR qPCR mix, 1 µl each primer (50 µM stock), 7 µl water and 1 µl cDNA, were amplified under the following thermal cycling conditions: 95 °C treatment for 2 min; 45 cycles of 10 s at 95 °C and 20 s at 55 °C; and incubation for 20 s at 72 °C. The expression levels of 16S rRNA were used for the normalization of the levels of the test samples, and the relative expression levels were quantified using Relative Quantification software (Roche). Results are presented as the mean values of three independent experiments.

### Western blot analysis

Western blot analysis for PAGE gels was carried out by a standard method as described previously, with some modification [37]. After the gel-shift assay, gels were blotted onto PVDF membrane using a semi-dry transfer apparatus. Proteins on the membranes were immuno-detected with anti-RpoN or anti-RpoD antibodies, and then detected with ImmunoStar Zeta (Fujifilm). Images were analysed with LuminoGraph I (Atto).

## Results and Discussion

### gSELEX screening *in vitro*

Transcription by the RNAP RpoN holoenzyme is believed to depend on an enhancer (or activator), which promotes the transition from a closed promoter complex to an open complex for transcription initiation. This scenario was established using a single species of the enhancer NtrC [8–11]. To identify the whole set of RpoN-dependent promoters in the entire genome of *E. coli* K-12 W3110, and to identify the role of each enhancer, we performed a mass-screening *in vitro* of the whole set of sequences that are recognized by the reconstituted RNAP RpoN holoenzyme and the well-characterized NtrC as a model system. The sigma-free core enzyme was prepared by passing the purified RNAP (stored in a storage buffer containing 50% (v/v) glycerol) through a phosphocellulose chromatography column, three times in the presence of 5% (v/v) glycerol [35]. The level of remaining sigma subunits, if any, was less than 0.1 %, as detected by both protein staining and immunostaining against each of the seven sigma factor species (RpoD, RpoN, RpoS, RpoH, RpoF, RpoE and FecI). The stoichiometry between core enzyme subunits was also checked by immunostaining with antibodies against each core subunit, RpoA, RpoB, RpoC and RpoZ. The holoenzymes fully saturated with each sigma subunit were reconstituted by mixing this sigma-free core enzyme and fourfold molar excess of purified RpoN sigma factor. As these sigma subunits alone are unable to bind to promoter DNA, the presence of excess sigma does not interfere with the function of RNAP holoenzymes. For the identification of DNA sequences that are recognized by each holoenzyme, we employed the gSELEX screening system [24], in which a library of *E. coli* genomic DNA fragments of 200–300 bp was used instead of synthetic oligonucleotides with all possible sequences used in the original SELEX method [41–43].

A multi-copy plasmid library of 200–300 bp random DNA fragments was constructed from the *E. coli* K-12 W3350 genome [23, 24]. The library used in this study contained 5.5-fold molar excess of the entire genome and, thus, a single and the same sequence may be included in five different overlapping segments on average, thereby increasing the resolution of SELEX fragment mapping. In each experiment of gSELEX screening, the mixture of genomic DNA fragments, which was regenerated by PCR from the genomic DNA library, was mixed with a twofold molar excess of the reconstituted RpoN holoenzyme and subjected to gSELEX screening. The DNA–holoenzyme complexes formed were recovered using the anti-RpoC antibody, which gave the highest level of RNAP recovery among all the anti-core subunit antibodies. RNAP-associated DNA was isolated from the antibody precipitates, amplified by PCR and subjected to cycles of gSELEX. After repeated gSELEX screening, the final products of RpoN holoenzyme-bound DNA fragments were mapped onto the genome using a DNA tiling microarray (Oxford Gene Technology) [44]. The binding intensity was measured as the ratio of RpoN holoenzyme-bound DNA labelled with Cy3 against the original library DNA labelled with Cy5 on an array, and plotted along the *E. coli* genome for each holoenzyme. In the case of NtrC, His-tagged NtrC was purified and the binding reaction with the genomic DNA library was performed in the presence of acetylphosphate for NtrC activation by phosphorylation [45]. The NtrC-associated DNA was isolated using a Ni-NTA agarose affinity column and subjected to DNA tiling array analysis. During the DNA tiling array, 60 bp long probes were aligned along the *E. coli* genome at 105 bp intervals; therefore, approximately 300 bp long gSELEX fragments were bound to two or more consecutive probes. This criterion was employed to avoid background noise of the non-specific binding of holoenzyme-bound DNA fragments to the tiling array; it is worth noting that peaks showing hybridization to only a single probe were judged as false-positive noise.

Binding sites were classified into two groups: one ‘within spacers’ and the other ‘inside genes’. Binding sites of the within spacers group were further classified into three types: a type-A spacer, located between bidirectional transcription units; a type-B spacer, located upstream of one transcription unit but downstream of another transcription unit; a type-C spacer, located downstream of both transcription units. Based on the transcription direction of flanking genes, the total number of constitutive promoters was predicted to range between the minimum (number of type-A spacers+number of type-B spacers) and maximum (number of type-A spacers×2+number of type-B spacers). The height of the binding intensity identified by the gSELEX-chip system is generally in good agreement with the number of clones identified by the gSELEX-clos (cloning-sequencing) system, indicating that these two parameters correlate with the binding affinity of the test regulatory protein to DNA [24].

### Identification of the whole set of constitutive promoters recognized by the RpoN holoenzyme alone

**Fig. 1.**
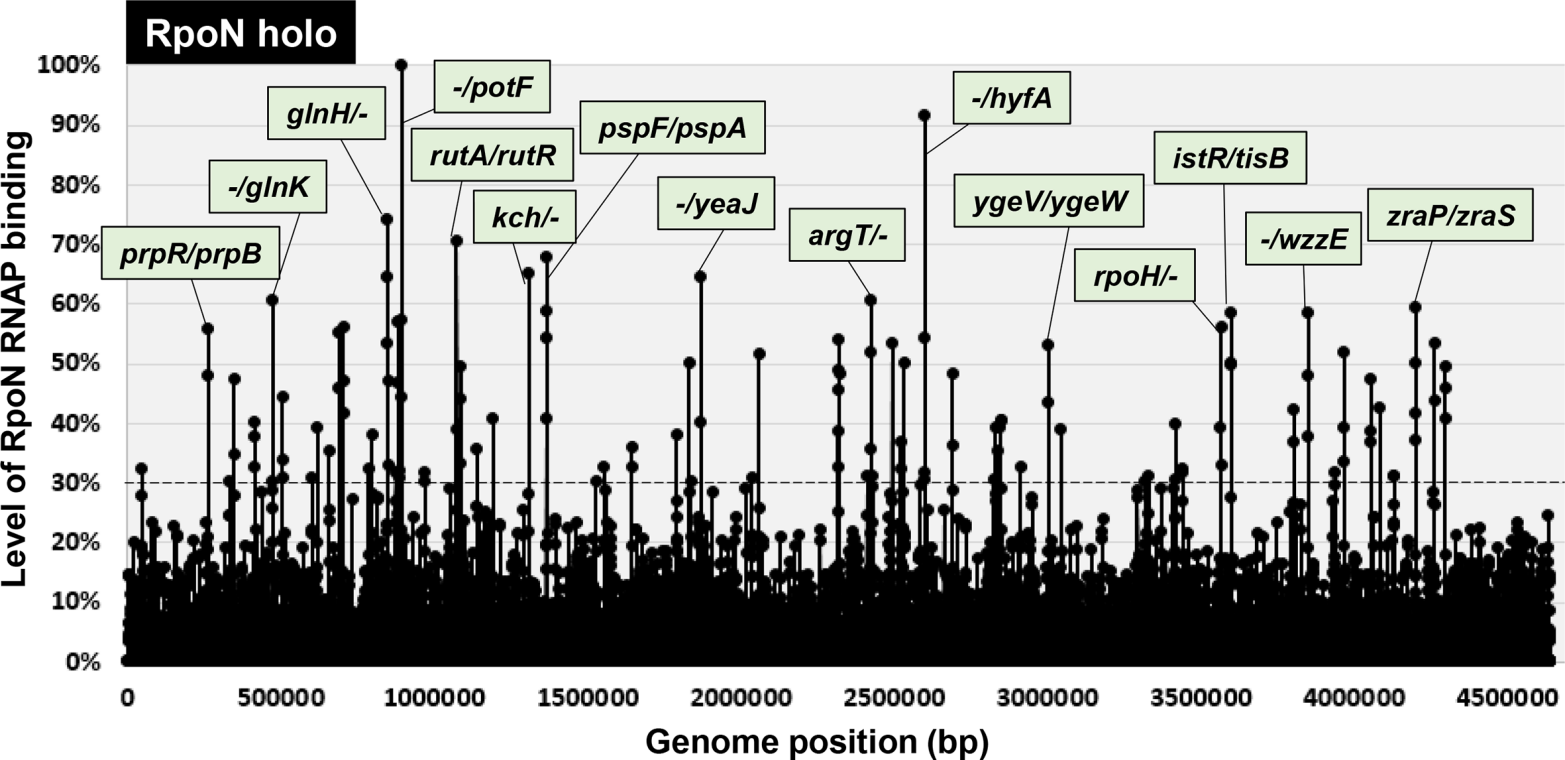
gSELEX-chip search for the binding sequences of the RpoN RNAP holoenzyme on the *E. coli* K-12 genome. gSELEX was performed to search for the binding sites of the RNAP RpoN holoenzyme. The *y*-axis represents the ratio against the highest peak at the *potF* promoter region and shows the level of RpoN holoenzyme-bound DNA fragments, whereas the *x*-axis represents the position on the *E. coli* K-12 genome in bp. The adjacent gene on the *E. coli* K-12 genome of the peak position was indicated for high intensity peaks (>60%). A list of binding sites of the RpoN holoenzyme is provided in Table 1 (detailed in Table S2).

To identify the whole set of RpoN recognition promoters, we performed gSELEX screening for the RpoN RNAP holoenzyme. After seven cycles of gSELEX screening, the sequences with a binding affinity to the RpoN holoenzyme formed a number of peaks along the entire *E. coli* genome (Fig. 1). By setting the cut-off level to 30% relative to the highest peak located upstream of *potF* (putrescine transporter), a total of 71 RpoN holoenzyme-binding peaks were identified, of which 44 (62%) were located within intergenic spacers (Fig. 1; detailed in Table S2), in addition to 17 peaks inside type-A spacers and 27 peaks inside type-B spacers (Table S2). From the RpoN holoenzyme-binding sites inside type-A and type-B spacers, a total of 44 (17 type-A+27 type-B) to 61 (17×2 type A+27 type B) promoters were tentatively identified as constitutive promoters recognized by the RpoN holoenzyme (Table 1). A total of 27 peaks (38 %) were located inside the ORFs (Fig. 1; detailed in Table S2).

**Table 1.** RpoN holoenzyme-binding sites on the *E. coli* K-12 genome gSELEX was performed to search for binding sites of the RpoN RNAP holoenzyme. By setting the cut-off level to 30%, a total of 71 binding sites were identified (see Fig. 1 for gSELEX pattern), which have been aligned along the map of the *E. coli* K-12 genome. Binding intensity of the RNAP RpoN holoenzyme is shown in the RpoN holo column (see Table S2; the dark orange shading shows the intensity 61–100%, medium orange shading shows 41–60% and pale orange shading shows 30–40%). A total of 44 sites are located within intergenic spacers: 17 within type-A spacers and 27 within type-B spacers (see Table 2). Columns D indicate the direction of transcription. The potential target genes or operons of RpoN were predicted based on the adjacent genes and the gene orientation (shown with green shading). The grey shading shows genes that are not potential targets.

No.	gSELEX peak type	Map position (bp)	RpoN holo	Left gene function	Operon	Left gene	D	RpoN holo	D	Right gene	Operon	Right gene function
1	A	347864	47%	DNA-binding transcriptional activator	*prpR*	*prpR*	<		>	*prpB*	*prpBCDE*	2-Methylisocitrate lyase
2	B	471846	61%			*mdlB*	>		>	*glnK*	*glnK-amtB*	Nitrogen assimilation regulatory protein for GlnL, GlnE and AmtB
3	B	619432	39%	Iron-enterobactin transporter subunit	*fepC*	*fepC*	<		<	*fepG*		
4	A	655760	35%	Anaerobic C4-dicarboxylate transport	*dcuC*	*dcuC*	<		>	*pagP*	*pagP*	Palmitoyl transferase for lipid A
5	B	688560	55%	IS*5* transposase and trans-activator	*insH*	*insH*	<		<	*lnt*		
6	A	784656	32%	Conserved protein	*ybgS*	*ybgS*	<		>	*aroG*	*aroG*	3-Deoxy-d-arabino-heptulosonate-7-phosphate synthase
7	B	847362	74%	Glutamine transporter subunit	*glnHPQ*	*glnH*	<		<	*dps*		
8	B	874568	32%			*yliE*	>		>	*yliF*	*yliF*	Predicted diguanylate cyclase
9	A	882830	57%	Undecaprenyl pyrophosphate phosphatase	*ybjG*	*ybjG*	<		>	*cmr*	*cmr*	Multidrug efflux system protein
10	B	891170	44%			*nfsA*	>		>	*rimK*	*rimK-ybjN*	Ribosomal protein S6 modification protein
11	B	892632	100 %			*ybjN*	>		>	*potF*	*potFGHI*	Putrescine transporter subunit: periplasmic-binding component of ABC superfamily
12	A	1073268	71%	Predicted monooxygenase	*rutABCDEFG*	*rutA*	<		>	*rutR*	*rutR*	Predicted DNA-binding transcriptional regulator
13	B	1191232	41%	Adenylosuccinate lyase	*purB*	*purB*	<		<	*hflD*		
14	B	1308556	65%	Voltage-gated potassium channel	*kch*	*kch*	<		<	*yciI*		
15	A	1366070	68%	DNA-binding transcriptional activator	*pspF*	*pspF*	<		>	*pspA*	*pspABCDE*	Regulatory protein for phage-shock-protein operon
16	B	1527534	30%			*yncH*	>		>	*ydcD*	*ydcD*	Predicted protein
17	A	1830436	50%	Succinylornithine transaminase, PLP-dependent	*astCADBE*	*astC*	<		>	*xthA*	*xthA*	Exonuclease III
18	A	2036832	31%	Predicted DNA-binding response regulator in TCS with YedV	*yedWV*	*yedW*	<		>	*hiuH*	*hiuH*	Hydroxyisourate hydrolase/transthyretin-related protein
19	A	2060070	52%	DNA-binding transcriptional dual regulator of nitrogen assimilation	*nac*	*nac*	<		>	*asnV*	*asnV*	Asn tRNA
20	B	2321470	48%			*atoC*	>		>	*atoD*	*atoDAEB*	Acetyl-CoA:acetoacetyl-CoA transferase, alpha subunit
21	A	2411432	31%	Conserved inner membrane protein	*yfbV*	*yfbV*	<		>	*ackA*	*ackA-pta*	Acetate kinase A and propionate kinase 2
22	B	2425832	61%	Lysine/arginine/ornithine transporter subunit	*argT-hisJQMP*	*argT*	<		<	*ubiX*		
23	B	2429072	31%	Membrane protein required for colicin V production	*cvpA-purF-ubiX*	*cvpA*	<		<	*dedD*		
24	A	2493362	54 %	Predicted inner membrane protein	*yfdY*	*yfdY*	<		>	*lpxP*	*lpxP*	Palmitoleoyl-acyl carrier protein-dependent acyltransferase
25	B	2520564	32%	DNA-binding transcriptional activator	*xapR*	*xapR*	<		<	*xapB*		
26	B	2531464	50%			*cysK*	>		>	*ptsH*	*ptsHI-crr*	Phosphohistidinoprotein-hexose phosphotransferase component of PTS system
27	B	2599140	92%			*bcp*	>		>	*hyfA*	*hyfABCDEFGHIJR-focB*	Hydrogenase 4, 4Fe-4S subunit
28	B	2689364	48%	ncRNA	*glmY*	*glmY*	<		<	*purL*		
29	B	2825748	31%			*srlB*	>		>	*srlD*	*srlD-gutM-srlR-gutQ*	Sorbitol-6-phosphate dehydrogenase
30	B	2836270	35%	Formate dehydrogenase-H, [4Fe-4S] ferredoxin subunit	*hydN-hypF*	*hydN*	<		<	*ascG*		
31	A	2848650	40%	Regulator of the transcriptional regulator FhlA	*hycABCDEFGHI*	*hycA*	<		>	*hypA*	*hypABCDE-fhlA*	Protein involved in nickel insertion into hydrogenases 3
32	A	3004270	53%	Predicted DNA-binding transcriptional regulator	*ygeV*	*ygeV*	<		>	*ygeW*	*ygeW*	Conserved protein
33	B	3043930	39%	Predicted NAD(P)-binding oxidoreductase with NAD(P)-binding Rossmann-fold domain	*ygfF*	*ygfF*	<		<	*gcvP*		
34	B	3417032	40%			*yhdV*	>		>	*yhdX*	*yhdXYZ*	Predicted amino-acid transporter subunit
35	B	3440634	32%	30S ribosomal subunit protein S13	*rpsMKD-rpoA-rplQ*	*rpsM*	<		<	*rpmJ*		
36	B	3598870	59%	RNAP sigma 32 (sigma H) factor	*rpoH*	*rpoH*	<		<	*ftsX*		
37	A	3851352	59%	ncRNA	*istR*	*istR*	<		>	*tisB*	*tisB*	LexA-regulated toxic peptide
38	B	3967058	52%			*rfe*	>		>	*wzzE*	*wzzE-wecBC-rffGHCA-wzxE-rffT-wzyE-rffM*	Entobacterial common antigen polysaccharide chain length modulation protein
39	A	4056244	47%	Glutamine synthetase	*glnALG*	*glnA*	<		>	*typA*	*typA*	GTP-binding protein
40	A	4083972	43%	Formate dehydrogenase-O, large subunit	*fdoGHI-fdhE*	*fdoG*	<		>	*fdhD*	*fdhD*	Formate dehydrogenase formation protein
41	B	4131538	31%			*metF*	>		>	*katG*	*katG*	Catalase/hydroperoxidase HPI(I)
42	A	4199860	60%	Zn-binding periplasmic protein	*zraP*	*zraP*	<		>	*zraS*	*zraSR*	Sensory histidine kinase in two-component regulatory system with ZraR
43	B	4260864	54%			*dusA*	>		>	*pspG*	*pspG*	Phage shock protein G
44	B	4297530	50%	Formate dehydrogenase-H, selenopolypeptide subunit	*fdhF*	*fdhF*	<		<	*mdtP*		
	**A=17,** **B=27**	**Cut-off**	**>30%**			**Constitutive promoters=44–61**						
		**Spacer**	**44**									

Of the 71 binding targets of the RpoN holoenzyme, 23 sites (32 %) were listed as RpoN targets in the RegulonDB database (RegulonDB column in Table S2). In contrast, 30 sites (42 %) were detected by ChIP-chip analysis (ChIP-chip column in Table S2) [17]. A total of 39 (55 %) were newly identified as RpoN targets in the *E. coli* genome in this study (see below).

### Identification of the whole set of NtrC target genes

**Fig. 2.**
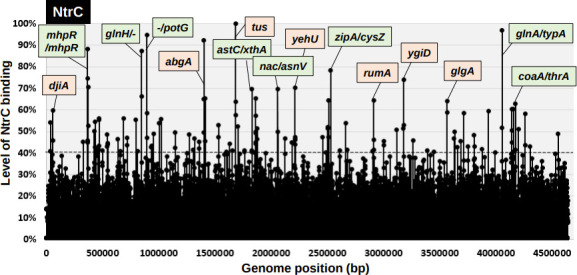
gSELEX-chip search for the binding sequences of NtrC on the *E. coli* K-12 genome. gSELEX was performed to search for the binding sites of NtrC, in the presence of acetylphosphate, with respect to NtrC phosphorylation. The *y*-axis represents the ratio against the highest peak at the *tus* ORF and shows the level of NtrC-bound DNA fragments, whereas the *x*-axis represents the position on the *E. coli* K-12 genome in bp. The adjacent gene on the *E. coli* K-12 genome of the peak position was indicated for high intensity peaks (>70%). Peaks located within the spacer regions are shown with green labels, while peaks located within ORFs are shown with orange labels. A list of the binding sites of NtrC is provided in Table 2 (detailed in Table S3).

NtrC was isolated as the nitrogen assimilation regulator encoded by *glnG* [46], and is known as an enhancer for nitrogen assimilation under nitrogen-limited conditions [47]. To understand genome regulation by NtrBC TCS in *E. coli *, we attempted to identify the whole set of regulatory target promoters, genes and operons under the control of phosphorylated NtrC. For this purpose, we independently employed gSELEX screening using purified His-tagged NtrC in the presence of 10 mM acetylphosphate for NtrC phosphorylation *in vitro*. From a mixture of *E. coli* K-12 W3110 genome fragments, NtrC-bound DNA fragments were affinity-purified using Ni-NTA agarose and then subjected to tiling array analysis to identify NtrC recognition sequences. This gSELEX screening was repeated for up to five cycles. The original mixture of genomic DNA fragments formed smeared bands during PAGE. However, after repeated gSELEX screening, the NtrC-bound DNA formed sharper bands during PAGE, indicating the enrichment of specific DNA fragments with specific binding activity to NtrC. Here, we identified a total of 93 high-intensity peaks by setting the cut-off level above an intensity of 40 %, relative to the highest peak located on the *tus* ORF (Fig. 2; for details, see Table S3). Of these 93 high-level binding peaks, seven binding sites are listed as NtrC target genes or operons in the RegulonDB database (RegulonDB column in Table S3). A total of 31 (33 %) NtrC-binding sites were found within the spacers, while 62 (67 %) were found inside the ORFs (Table S3). Of these 31 NtrC-binding sites within spacers, 11 were located within spacers of bidirectional transcription units (Table 2), 19 were located inside spacers upstream of one ORF but downstream of another ORF (Table 2), and two were located inside the type-C spacer (Table 2). Using these results, we predicted that the total number of regulatory targets of NtrC was between 30 (11 type A+19 type B) and 41 (11×2 type A+19×1 type B). We performed gSELEX screening for approximately 200 species of *E. coli* K-12 TFs. Although the binding of TFs inside ORFs is variable between TF species, the level of 67 % binding of NtrC inside ORFs was rather high, and its unidentified regulatory roles should be analysed in detail.

**Table 2.** NtrC-binding sites on the *E. coli* K-12 genome gSELEX was performed to search for the binding sites of NtrC. By setting the cut-off level to 40%, a total of 93 binding sites were identified (see Fig. 2 for gSELEX pattern), which are aligned along the map of the *E. coli* K-12 genome. Binding intensity of NtrC is shown in the NtrC column (the dark orange shading shows the intensity 61–100%, medium orange shading shows 41–60% and pale orange shading shows 30–40%). A total of 32 sites are located within intergenic spacers: 11 within type-A spacers; 19 within type-B spacers; 2 within type-C spacers (see Table S3). Columns D indicate the direction of transcription. Potential target genes or operons of NtrC were predicted based on the adjacent genes and gene orientation (shown with green shading). The grey shading shows genes that are not potential targets.

No.	gSELEX peak type	Map position (bp)	NtrC	Left gene function	Operon	Left gene	D	NtrC	D	Right gene	Operon	Right gene function
1	A	367 650	88%	DNA-binding transcriptional activator, 3HPP-binding	*mhpR-lacI*	*mhpR*	<		>	*mhpA*	*mhpABCDFE*	3-(3-Hydroxyphenyl)propionate hydroxylase
2	B	371 336	71%			*mhpC*	>		>	*mhpD*	*mhpDFE*	2-Keto-4-pentenoate hydratase
3	B	433 872	43%			*ribD*	>		>	*ribE*	*ribE-nusB-thiL-pgpA*	Riboflavin synthase beta chain
4	A	443 846	41%	2-Dehydropantoate reductase, NADPH-specific	*panE-yajL*	*panE*	<		>	*yajQ*	*yajQ*	Predicted nucleotide binding protein
5	B	471 846	56%			*mdlB*	>		>	*glnK*	*glnK-amtB*	Nitrogen assimilation regulatory protein for GlnL, GlnE and AmtB
6	B	547 672	44%			*fdrA*	>		>	*ylbF*	*ylbF-ybcF*	Conserved protein
7	A	655 760	41%	Anaerobic C4-dicarboxylate transport	*dcuC*	*dcuC*	<		>	*pagP*	*pagP*	Palmitoyl transferase for lipid A
8	B	688 560	56%	IS*5* transposase and trans-activator	*insH*	*insH*	<		<	*lnt*		
9	B	847 362	87%	Glutamine transporter subunit	*glnHPQ*	*glnH*	<		<	*dps*		
10	B	894 130	95%			*potF*	>		>	*potG*	*potGHI*	Putrescine transporter subunit: ATP-binding component of ABC superfamily
11	A	1 250 156	40%	Dihydroxyacetone kinase, N-terminal domain	*dhaKLM*	*dhaK*	<		>	*dhaR*	*dhaR*	Predicted DNA-binding transcriptional regulator, dihydroxyacetone
12	B	1 308 556	42%	Voltage-gated potassium channel	*kch*	*kch*	<		<	*yciI*		
13	B	1 613 766	40%			*yneJ*	>		>	*yneK*	*yneK*	Predicted protein
14	A	1 630 062	44%	Predicted mannonate dehydrogenase	*ydfI*	*ydfI*	<		>	*ydfK*	*ydfK*	Qin prophage; predicted DNA-binding transcriptional regulator
15	B	1 653 158	41%	Predicted dehydratase	*rspAB*	*rspA*	<		<	*ynfA*		
16	A	1 830 436	70%	Succinylornithine transaminase, PLP-dependent	*astCADBE*	*astC*	<		>	*xthA*	*xthA*	Exonuclease III
17	B	1 863 654	50%	Predicted oxidoreductase	*yeaE*	*yeaE*	<		<	*mipA*		
18	A	2 060 070	50%	DNA-binding transcriptional dual regulator of nitrogen assimilation	*nac*	*nac*	<		>	*asnV*	*asnV*	Asn tRNA
19	B	2 184 766	45%			*rcnA*	>		>	*rcnB*	*rcnB*	Periplasmic protein involved in nickel/cobalt export
20	A	2 458 968	47%	Conserved protein	*yfcZ*	*yfcZ*	<		>	*fadL*	*fadL*	Long-chain fatty acid outer membrane transporter
21	A	2 529 354	78%	Cell division protein involved in Z ring assembly	*zipA*	*zipA*	<		>	*cysZ*	*cysZ*	Predicted inner membrane protein
22	B	3 001 538	44%			*xdhB*	>		>	*xdhC*	*xdhC*	Xanthine dehydrogenase, Fe-S binding subunit
23	B	3 446 170	40%	50S ribosomal subunit protein L14	*rplNXE-rpsNH-rplFR-rpsE-rpmD-rplO-secY-rpmJ*	*rplN*	<		<	*rpsQ*		
24	B	3 933 336	59%			*rbsA*	>		>	*rbsC*	*rbsCBKR*	d-Ribose transporter subunit
25	B	3 994 336	40%			*yigA*	>		>	*xerC*	*xerC-yigB*	Site-specific tyrosine recombinase
26	A	4 056 244	97%	Glutamine synthetase	*glnALG*	*glnA*	<		>	*typA*	*typA*	GTP-binding protein
27	A	4 173 336	63%	Pantothenate kinase	*coaA*	*coaA*	<		>	*thrU*	*thrU-tyrU-glyT-thrT-tufB*	Thr tRNA
28	B	4 260 864	58%			*dusA*	>		>	*pspG*	*pspG*	Phage shock protein G
29	B	4 304 842	43%	Predicted alkyl sulfatase	*yjcS*	*yjcS*	<		<	*alsK*		
30	B	4 530 150	40%	KpLE2 phage-like element; predicted endoglucanase with Zn-dependent exopeptidase domain	*sgcXBCQAER*	*sgcX*	<		<	*yjhP*		
	**A=11,** **B=19**	**Cut-off**	**>40%**			**NtrC targets=30–41**						
		**Spacer**	**30**									

### Identification of the whole set of promoters recognized by NtrC-controlled RpoN holoenzyme

**Fig. 3.**
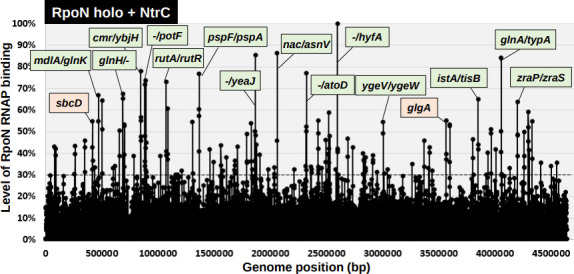
gSELEX-chip search for the binding sequences of the RpoN RNAP holoenzyme in the presence of NtrC on the *E. coli* K-12 genome. gSELEX was performed to search for binding sites of the RpoN holoenzyme in the presence of NtrC. The *y*-axis represents the ratio against the highest peak at the *hyfA* promoter region and shows the level of RpoN holoenzyme-bound DNA fragments in the presence of NtrC, whereas the *x*-axis represents the position on the *E. coli* K-12 genome in bp. The adjacent gene on the *E. coli* K-12 genome of the peak position was indicated for high intensity peaks (>60 %). The peaks located within the spacer regions are shown with green labels, while the peaks located within the ORFs are shown with orange labels. A list of the binding sites of the RpoN holoenzyme in the presence of NtrC is described in Table 3 (detailed in Table S4).

**Fig. 4.**
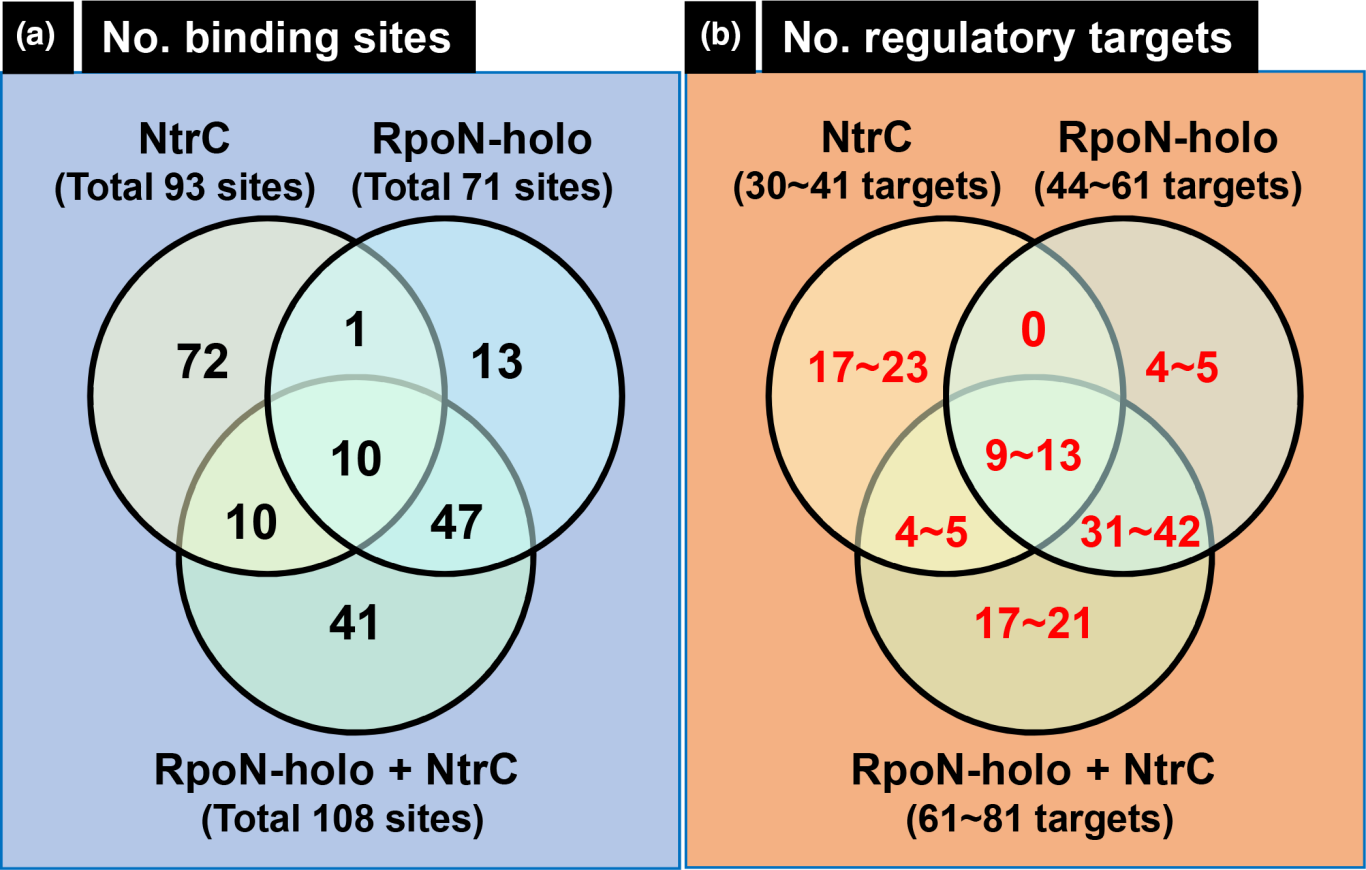
Correlation diagrams of the targets between RpoN and NtrC. Venn diagram summarizing the correlation of target sites of RpoN holoenzyme and NtrC. The number of binding sites is shown in (**a**), while the number of regulatory targets is shown in (**b**). All the 14 sites detected in the RpoN holoenzyme but not in RpoN holoenzyme+NtrC showed over 24% intensity in RpoN holoenzyme+NtrC (for counting the number of targets, the cut-off level was set as 30%) (Table 5).

For transcription initiation by the RpoN holoenzyme, one of the NtrC- or TyrR-family TFs, such as NtrC, is believed to be necessary [8–11]. To understand the intrinsic role of the RpoN sigma factor, we performed gSELEX screening of the regulatory target promoters of the RpoN holoenzyme in the presence of an excess amount of NtrC under the same conditions used for the screening of constitutive promoters by the RpoN holoenzyme alone. After seven cycles of gSELEX screening, RpoN holoenzyme-bound DNA segments were isolated using anti-RpoC antibody and then subjected to tiling array analysis. By setting the cut-off level to 30% relative to the highest peak located upstream of *hyfA* (subunit of hydrogenase 4), a total of 108 NtrC-controlled RpoN holoenzyme-binding peaks were identified (Fig. 3), of which 61 peaks (56 %) were located within intergenic spacers and 47 peaks (44 %) were located inside the ORFs (for details see Table S4). Of the 61 RpoN holoenzyme-binding peaks within spacers, 19 peaks were located within type-A spacers of bidirectional transcription units (Table 3, type-A), and 42 were located inside type-B spacers upstream of one transcription unit but downstream of another transcription unit (Table 3, type-B). The promoters recognized by a combination of the RpoN holoenzyme and NtrC were predicted to be located in the type-A and type-B spacers (Table 3). Of the 108 NtrC-controlled RpoN holoenzyme-binding sites, 57 sites were detected in the absence of NtrC (Table 4, Fig. 4a), suggesting that these promoters (44 to 61) could be recognized by the RpoN holoenzyme alone without the support of NtrC (Fig. 4b). This finding indicates the presence of constitutive promoters for RpoN sigma, as in the case of the other six sigma factors [26, 27]. In contrast, a total of 21 to 27 promoters required NtrC for binding to the target promoters (Fig. 4b), of which 4 to 5 promoters were recognized by NtrC alone, while the other 17 to 22 promoters were recognized by a combination of RpoN and NtrC (Fig. 4b).

**Table 3.** RpoN holoenzyme-binding sites in the presence of NtrC on the *E. coli* K-12 genome gSELEX was performed to search for binding sites of the RNAP RpoN holoenzyme in the presence of NtrC. By setting the cut-off level to 30%, a total of 108 binding sites were identified (see Fig. 3 for gSELEX pattern), which are aligned along the map of the *E. coli* K-12 genome. Binding intensity of NtrC is shown in the RpoN holo+NtrC column (the dark orange shading shows the intensity 61–100%, medium orange shading shows 41–60% and pale orange shading shows 30–40%). A total of 61 sites are located within intergenic spacers: 19 within type-A spacers; 42 within type-B spacers (see Table S4). Columns D indicate the direction of transcription. Potential target genes or operons of RpoN in the presence of NtrC were predicted based on the adjacent genes and gene orientation (shown with green shading). The grey shading shows genes that are not potential targets.

No.	gSELEX peak type	Map position (bp)	RpoN holo+NtrC	Left gene function	Operon	Left gene	D	RpoN+NtrC	D	Right gene	Operon	Right gene function
1	A	42 372	30%	Predicted transporter	*caiTABCDE*	*caiT*	<		>	*fixA*	*fixABCX*	Predicted electron transfer flavoprotein subunit, ETFP adenine nucleotide-binding domain
2	A	77 346	43%	DNA-binding transcriptional regulator	*sgrR-sroA-tbpA-thiPQ*	*sgrR*	<		>	*sgrS*	*sgrST-setA*	ncRNA
3	B	257 850	30%			*frsA*	>		>	*crl*	*crl*	DNA-binding transcriptional regulator
4	A	347 864	43%	DNA-binding transcriptional activator	*prpR*	*prpR*	<		>	*prpB*	*prpBCDE*	2-Methylisocitrate lyase
5	B	418 832	37%			*phoR*	>		>	*brnQ*	*brnQ-proY*	Predicted branched chain amino acid transporter (LIV-II)
6	B	471 846	67%			*mdlB*	>		>	*glnK*	*glnK-amtB*	Nitrogen assimilation regulatory protein for GlnL, GlnE and AmtB
7	B	619 432	34%	Iron-enterobactin transporter subunit	*fepC*	*fepC*	<		<	*fepG*		
8	A	655 760	50%	Anaerobic C4-dicarboxylate transport	*dcuC*	*dcuC*	<		>	*pagP*	*pagP*	Palmitoyl transferase for lipid A
9	B	688 560	67%	IS*5* transposase and trans-activator	*insH*	*insH*	<		<	*lnt*		
10	A	784 656	39 %	Conserved protein	*ybgS*	*ybgS*	<		>	*aroG*	*aroG*	3-Deoxy-d-arabino-heptulosonate-7-phosphate synthase, phenylalanine repressible
11	B	847 362	78%	Glutamine transporter subunit	*glnHPQ*	*glnH*	<		<	*dps*		
12	A	882 830	72%	Undecaprenyl pyrophosphate phosphatase	*ybjG*	*ybjG*	<		>	*cmr*	*cmr*	Multidrug efflux system protein
13	B	891 170	32%			*nfsA*	>		>	*rimK*	*rimK-ybjN*	Ribosomal protein S6 modification protein
14	B	892 632	74 %			*ybjN*	>		>	*potF*	*potFGHI*	Putrescine transporter subunit: periplasmic-binding component of ABC superfamily
15	B	894 130	45%			*potF*	>		>	*potG*	*potGHI*	Putrescine transporter subunit: ATP-binding component of ABC superfamily
16	A	1 073 268	73%	Predicted monooxygenase	*rutABCDEFG*	*rutA*	<		>	*rutR*	*rutR*	Predicted DNA-binding transcriptional regulator
17	B	1 177 842	30%			*lolE*	>		>	*nagK*	*nagK-cobB*	*N*-Acetyl-d-glucosamine kinase
18	B	1 308 556	54%	Voltage-gated potassium channel	*kch*	*kch*	<		<	*yciI*		
19	A	1 366 070	77%	DNA-binding transcriptional activator	*pspF*	*pspF*	<		>	*pspA*	*pspABCDE*	Regulatory protein for phage-shock-protein operon
20	B	1 561 132	44%	d-Ala-d-Ala dipeptidase, Zn-dependent	*ddpXABCDF*	*ddpX*	<		<	*dos*		
21	B	1 608 732	38%	Altronate oxidoreductase, NAD-dependent	*uxaB*	*uxaB*	<		<	*yneF*		
22	B	1 678 972	32%			*ydgI*	>		>	*folM*	*folM*	Dihydrofolate reductase isozyme
23	B	1 709 534	43%			*rsxE*	>		>	*nth*	*nth*	DNA glycosylase and apyrimidinic lyase (endonuclease III)
24	A	1 830 436	54%	Succinylornithine transaminase, PLP-dependent	*astCADBE*	*astC*	<		>	*xthA*	*xthA*	Exonuclease III
25	B	1 863 654	50%	Methylglyoxal reductase	*yeaE*	*yeaE*	<		<	*mipA*		
26	B	1 905 652	39%	Predicted protein	*yobF-cspC*	*yobF*	<		<	*yebO*		
27	A	2 036 832	33%	Predicted DNA-binding response regulator in TCS with YedV	*yedWV*	*yedW*	<		>	*hiuH*	*hiuH*	Hydroxyisourate hydrolase/transthyretin-related protein
28	A	2 060 070	86%	DNA-binding transcriptional dual regulator of nitrogen assimilation	*nac*	*nac*	<		>	*asnV*	*asnV*	Asn tRNA
29	B	2 321 470	77%			*atoC*	>		>	*atoD*	*atoDAEB*	Acetyl-CoA:acetoacetyl-CoA transferase, alpha subunit
30	B	2 360 468	34%	Predicted DNA-binding transcriptional regulator	*yfaX-rhmD-yfaVU*	*yfaX*	<		<	*yfaY*		
31	B	2 425 832	55%	Lysine/arginine/ornithine transporter subunit	*argT-hisJQMP*	*argT*	<		<	*ubiX*		
32	B	2 429 072	32%	Membrane protein required for colicin V production	*cvpA-purF-ubiX*	*cvpA*	<		<	*dedD*		
33	A	2 458 968	33%	Conserved protein	*yfcZ*	*yfcZ*	<		>	*fadL*	*fadL*	Long-chain fatty acid outer membrane transporter
34	A	2 493 362	46%	Predicted inner membrane protein	*yfdY*	*yfdY*	<		>	*lpxP*	*lpxP*	Palmitoleoyl-acyl carrier protein (ACP)-dependent acyltransferase
35	B	2 520 564	59%	DNA-binding transcriptional activator	*xapR*	*xapR*	<		<	*xapB*		
36	B	2 522 072	36%	Xanthosine transporter	*xapB*	*xapB*	<		<	*xapA*		
37	B	2 531 464	48%			*cysK*	>		>	*ptsH*	*ptsHI-crr*	Phosphohistidinoprotein-hexose phosphotransferase component of PTS system
38	B	2 599 140	100%			*bcp*	>		>	*hyfA*	*hyfABCDEFGHIJR-focB*	Hydrogenase 4, 4Fe-4S subunit
39	B	2 689 364	47%	ncRNA	*glmY*	*glmY*	<		<	*purL*		
40	B	2 825 748	40%			*srlB*	>		>	*srlD*	*srlD-gutM-srlR-gutQ*	Sorbitol-6-phosphate dehydrogenase
41	A	2 830 336	30%	DNA-binding transcriptional activator	*norR*	*norR*	<		>	*norV*	*norVW*	Flavorubredoxin oxidoreductase
42	B	2 836 270	36%	Formate dehydrogenase-H, [4Fe-4S] ferredoxin subunit	*hydN-hypF*	*hydN*	<		<	*ascG*		
43	A	2 848 650	38%	Regulator of the transcriptional regulator FhlA	*hycABCDEFGHI*	*hycA*	<		>	*hypA*	*hypABCDE-fhlA*	Protein involved in nickel insertion into hydrogenases 3
44	A	3 004 270	55%	Predicted DNA-binding transcriptional regulator	*ygeV*	*ygeV*	<		>	*ygeW*	*ygeW*	Conserved protein
45	B	3 043 930	33%	Predicted NAD(P)-binding oxidoreductase with NAD(P)-binding Rossmann-fold domain	*ygfF*	*ygfF*	<		<	*gcvP*		
46	B	3 370 654	46%	Sialic acid transporter	*nanTEK-yhcH*	*nanT*	<		<	*nanA*		
47	B	3 408 032	33%			*prmA*	>		>	*dusB*	*dusB-fis*	tRNA-dihydrouridine synthase B
48	B	3 417 032	43 %			*yhdV*	>		>	*yhdX*	*yhdXYZ*	Predicted amino-acid transporter subunit
49	B	3 440 634	32%	30S ribosomal subunit protein S13	*rpsMKD-rpoA-rplQ*	*rpsM*	<		<	*rpmJ*		
50	B	3 598 870	53%	RNAP sigma 32 (sigma H) factor	*rpoH*	*rpoH*	<		<	*ftsX*		
51	B	3 809 172	30%	Formamidopyrimidine/5-formyluracil/5-hydroxymethyluracil DNA glycosylase	*mutM*	*mutM*	<		<	*rpmG*		
52	A	3 851 352	65%	ncRNA	*istR*	*istR*	<		>	*tisB*	*tisB*	LexA-regulated toxic peptide
53	B	3 967 058	51%			*rfe*	>		>	*wzzE*	*wzzE-wecBC-rffGHCA-wzxE-rffT-wzyE-rffM*	Entobacterial common antigen polysaccharide chain length modulation protein
54	B	4 008 248	42%			*pldB*	>		>	*yigL*	*yigL*	Predicted hydrolase
55	A	4 056 244	84%	Glutamine synthetase	*glnALG*	*glnA*	<		>	*typA*	*typA*	GTP-binding protein
56	B	4 131 538	33%			*metF*	>		>	*katG*	*katG*	Catalase/hydroperoxidase HPI(I)
57	A	4 199 860	64 %	Zn-binding periplasmic protein	*zraP*	*zraP*	<		>	*zraS*	*zraSR*	Sensory histidine kinase in two-component regulatory system with ZraR
58	B	4 260 864	50%			*dusA*	>		>	*pspG*	*pspG*	Phage shock protein G
59	B	4 297 530	59%	Formate dehydrogenase-H, selenopolypeptide subunit	*fdhF*	*fdhF*	<		<	*mdtP*		
60	B	4 304 842	41%	Predicted alkyl sulfatase	*yjcS*	*yjcS*	<		<	*alsK*		
61	B	4 331 330	55%	Sensory histidine kinase in two-component regulatory system with BasR	*basS*	*basS*	<		<	*basR*		
	**A=19,** **B=42**	**Cut-off**	**>30%**			**Regulatory targets=61–80**						
		**Spacer**	**108**									

**Table 4.** Summary of binding sites of the RpoN holoenzyme and NtrC The binding site of each RpoN holoenzyme and NtrC on the *E. coli* K-12 W3110 genome was determined *in vitro* using the gSELEX screening system. Details of the experimental procedures are described in a previous study [23]. The number of the target transcription units was estimated based on the location of the binding sites

Regulator	Total no. of binding sites	Inside spacer			Inside ORF	No. of regulatory targets		
		Type-A	Type-B	Type-C		Type-A spacer	Type-B spacer	Total
RpoN-holo	71	17	27	0	27 (38%)	17–34	27	44–61
		Total 44 (62%)						
NtrC	93	11	19	2	61 (66%)	11–22	19	30–41
		Total 32 (34%)						
RpoN-holo+NtrC	108	20	41	0	47 (44%)	20–40	41	61–81
		Total 61 (56%)						

### Sequences recognized by the RpoN holoenzyme and NtrC

Using the RpoN-binding sequence from a small number of RpoN targets, a 17 bp long sequence consisting of conserved GG at the ‒24 site and GC at the ‒12 site was proposed as the RpoN promoter motif [6, 17], which is different from the well-known TTGACA (‒35) and TATAAT (‒10) promoter sequences of RpoD group sigma factors. The RpoN promoter motif was then re-evaluated using the entire set of 71 RpoN holoenzyme-binding targets (see Table 4, RpoN-holo row), which includes 32 known targets (Table S2). To identify the RpoN promoters within the binding sites of the RpoN holoenzyme, a collection of 500 bp sequences from 71 targets was analysed via *in silico* search using the meme program [38]. Subsequently, we identified a 15 bp long sequence, (‒24 side) TGGCACnnTTnTTGC (‒12 side) (Table S2), which included the proposed RpoN promoter motif TGC at the ‒12 bp site and TGGCA at the ‒24 bp site (Fig. 4a). Previous studies have performed promoter sequence prediction using the experimental data obtained *in vivo* for enhancer-dependent promoters. Therefore, this study is to our knowledge the first to analyse the promoter sequence recognized by the RpoN holoenzyme alone in the absence of supporting TFs.

**Fig. 5.**
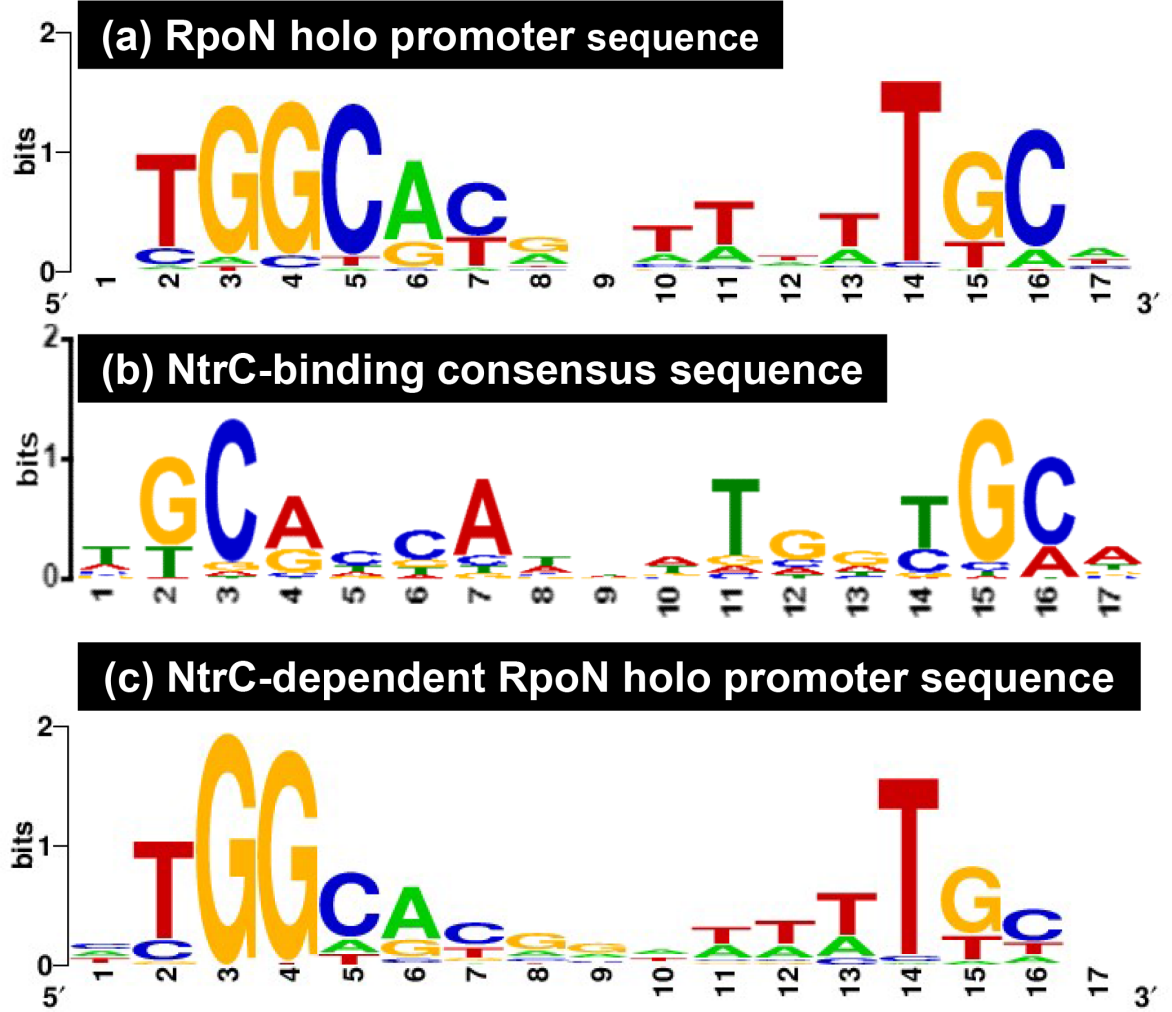
Consensus sequences of the RpoN holoenzyme promoter and NtrC binding. The promoter motif of RpoN holoenzyme, in the presence or absence of NtrC and binding sequences of NtrC, was analysed using the meme program. The sequences are listed in Tables S2–S4, and were subjected to Logo analysis for the determination of the consensus sequences for the following samples: (**a**) the whole set of RpoN holoenzyme targets (total 71 sequences in Table S2); (**b**) the whole set of NtrC targets (total 93 sequences in Table S3); (**c**) the whole set of RpoN holoenzyme targets in the presence of NtrC, not included in the RpoN holoenzyme targets in the absence of NtrC (total 51 sequences in Table S4).

Using the DNA-binding sequences of several NtrC targets, a 17 bp long palindromic sequence consisting of a 17 bp long sequence of TGCACCAnnnTGGTGCA was proposed as the consensus recognition sequence of NtrC [8]. As we obtained a large number of NtrC-binding sites by gSELEX, the consensus sequence of NtrC binding was re-evaluated using the whole set of 93 targets, including 7 known targets (Table 2). A collection of 500 bp sequences from these targets was analysed using the meme program. Subsequently, we identified a 17 bp long sequence (Fig. 5b), which contained highly conserved GCAnnA and TnnTGC. This sequence is in good agreement with a previous report using *in vitro* experimental evidence [8]. Thus, we concluded that this highly conserved (T)GCA(CC)AnnnT(GG)TGC(A) 17 bp long NtrC-box sequence is required for the tight binding of NtrC.

Finally, we analysed the promoter sequences recognized by the RpoN holoenzyme in the presence of excess NtrC. Some of the conserved sequences of promoters recognized by the RpoN holoenzyme alone were lost in the presence of NtrC, suggesting a certain level of alteration of the promoter recognition property in the presence of NtrC. Compared with the promoter sequence of the RpoN holoenzyme alone (Fig. 5a), the NtrC-dependent RpoN promoter sequence showed high-level conservation at the 3rd G and 4th G, but low-level conservation at the 5th, 15th and 16th C (Fig. 5c). These results suggest that NtrC modulates the promoter recognition property of the RpoN holoenzyme to recognize sequences of low-level conservation at the position of the ‒12 GC element.

**Fig. 6.**
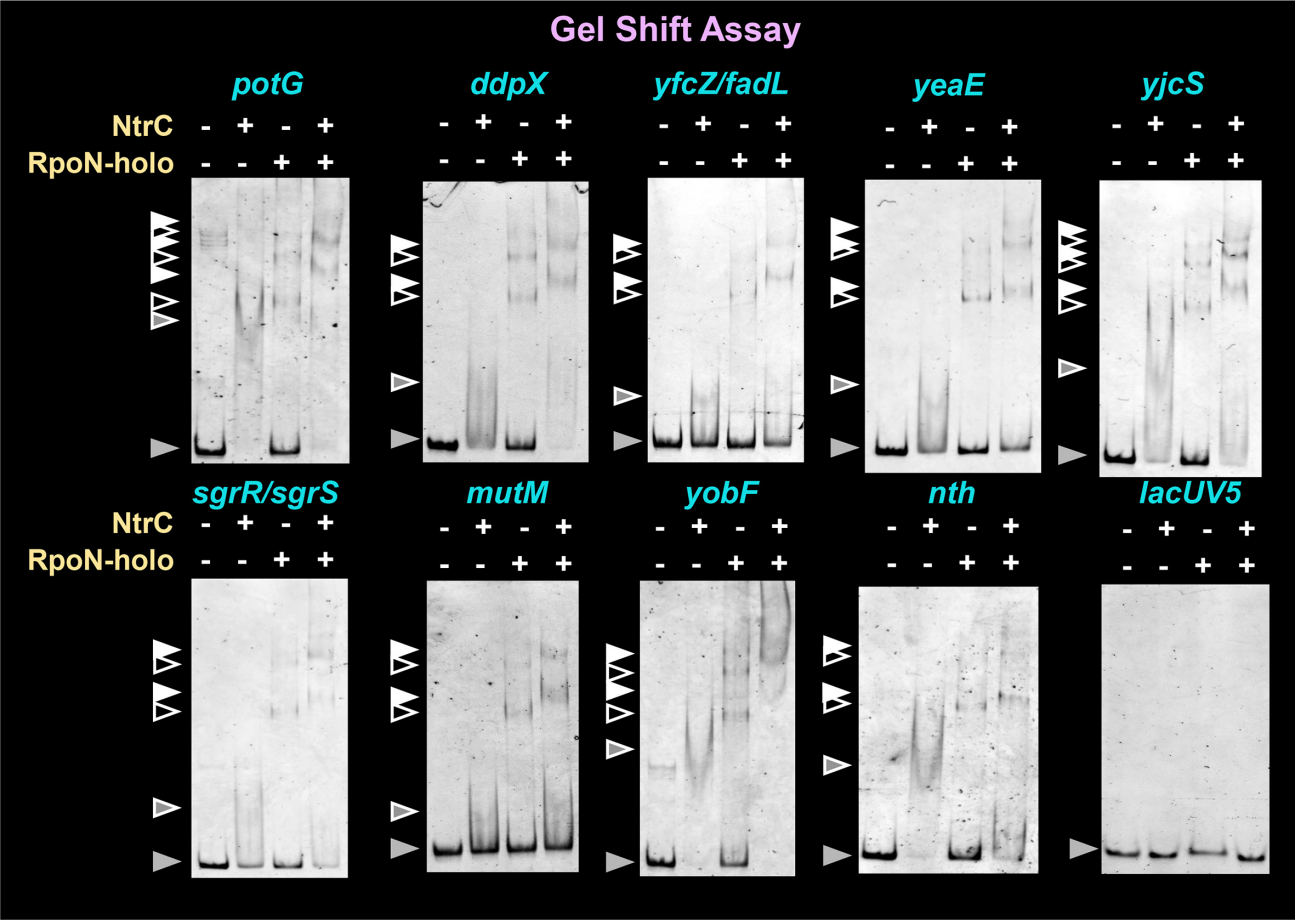
NtrC-dependent RpoN holoenzyme–DNA complex formation. The target promoter fragments were mixed with the RpoN holoenzyme (0.3 µM, lane 2), NtrC protein (15 µM, lane 3), or both in combination with the addition of 25 mM acetylphosphate (lane 4). After incubation at 37 °C for 30 min, the reaction mixture was subjected to 3.5% PAGE. Grey triangles indicate the free probe; grey triangles with white frame indicate the NtrC–probe complex; black triangles with white frame indicate the RpoN holoenzyme–probe complex; white triangles indicate the RpoN holoenzyme–NtrC-probe complex.

The RpoN holoenzyme binds to promoters with conserved sequence elements at −24 GG and −12 GC. One unique feature of the NtrC-dependent RpoN promoter identified in this study is the conservation of these elements, which was low for ‒12 GC and high for ‒24 GG (Fig. 5). This ‒12 GC element is involved in the stability of the RpoN holoenzyme–target promoter complex *in vitro* [48], while the ‒24 GG element is the dominant element for promoter binding by the RpoN holoenzyme [49]. In conjunction with the results of the gel-shift assay (Fig. 6), NtrC appears to support the stability of the formation of RpoN holoenzyme–promoter complex, which has a low-level conservation of the ‒12 GC element.

### Confirmation of the interaction of newly identified regulatory targets with RpoN holoenzyme and NtrC

#### Gel-shift assay *in vitro*

Based on gSELEX-chip analysis, we identified 44–61 promoters for the RpoN holoenzyme alone and 61–81 promoters for the NtrC-supported RpoN holoenzyme (Table 4, Fig. 4), including approximately 40 hitherto identified RpoN promoters. Similarly, we identified a total of 30–41 regulatory targets for NtrC via gSELEX screening (Table 4, Fig. 4). To experimentally confirm the regulation of newly identified target promoters with RpoN and/or NtrC, the interaction of the RpoN holoenzyme or NtrC with some representative targets was analysed by gel-shift assay *in vitro*, and RT-qPCR assay *in vivo*, of target mRNA.

To confirm the binding activity of both the RpoN holoenzyme and NtrC to the target promoters *in vitro*, we performed a gel-shift assay to detect the test protein–target DNA complexes. From the newly identified target genes, seven independent spacer probes containing nine representative targets were selected: *yfcZ* (uncharacterized conserved protein)/*fadL* (long-chain fatty acid outer membrane transporter), *yeaE* (aldo-keto reductase), *yjcS* (metallo-lactamase family protein), *sgrR* (TF)/*sgrS* (small RNA antisense regulator), *mutM* (DNA glycosylase), *yobF* (hypothetical protein) and *nth* (endonuclease III) (targets are indicated in red in Table 5). In addition, we used two hitherto known RpoN promoters, namely, *potG* encoding putrescine transporter and *ddpX* encoding d-Ala-d-Ala dipeptidase [12] (these two genes were also detected in gSELEX screening), and *lacUV5* as a reference control. Each of these test probes was mixed with purified NtrC, RpoN holoenzyme or both, and the probe–test protein(s) mixtures were then directly subjected to PAGE. The two known NtrC target probes, *potG* and *ddpX*, formed both NtrC–probe binary complexes and RpoN holoenzyme–probe binary complexes (Fig. 6; *potG* and *ddpX* panels, lanes 2 and 3). In both cases, two or three bands were detected, suggesting the presence of more than one promoter-like sequence on the *potG* and *ddpX* promoter probes. In the presence of both NtrC and the RpoN holoenzyme, the migration of the complex bands was significantly retarded, indicating the formation of RpoN holoenzyme–NtrC-probe ternary complexes (Fig. 6; *potG* and *ddpX* panels, lane 4). Next, we assessed seven spacer probes containing nine newly identified targets from the gel-shift assay under the same conditions as those employed for the two known targets. As in the case of *potG* and *ddpX* probes, the binding of NtrC alone (Fig. 6, lane 2) was confirmed by the disappearance of free probes. However, the expected probe–NtrC complex formed a smear band, likely due to the gradual dissociation of low-affinity NtrC during PAGE. In contrast, all seven DNA probes formed two or three detectable bands of RpoN holoenzyme–probe complexes (Fig. 6, lane 3). In the presence of both the RpoN holoenzyme and NtrC, the intensity of the free promoter probe clearly decreased for all seven probes, indicating an increase in DNA-binding intensity in the presence of both the RpoN holoenzyme and NtrC (Fig. 6, lanes 3 and 4). The simultaneous binding of the RpoN holoenzyme and NtrC was observed, based on the super shift of protein–DNA complexes from RpoN holoenzyme alone (lane 3) to the mixture of RpoN holoenzyme and NtrC (lane 4). These observations indicate the enhancement of RpoN holoenzyme binding to target promoters by NtrC. Neither the RpoN holoenzyme nor NtrC exhibited binding to a non-specific *lacUV5* promoter region used as an internal reference (Fig. 6, *lacUV5* panel).

**Table 5.** Summary of regulatory targets of RpoN holoenzyme, NtrC and RpoN holoenzyme–NtrC Binding sites and intensity of RpoN (Table 1), NtrC (Table 2) and RpoN–NtrC (Table 3) were combined (the dark orange shading shows the intensity 61–100%, medium orange shading shows 41–60% and pale orange shading shows 30–40%). The known regulatory targets of RpoN and NtrC in the RegulonDB database are shown in the Regulon RpoN and Regulon NtrC columns, respectively (shown in yellow). RpoN targets detected by ChIP-chip analysis are shown in the ChIP-chip column (shown in blue). Columns D indicate the direction of transcription. Potential target genes or operons of RpoN and/or NtrC were predicted based on the adjacent genes and gene orientation (shown with green shading). The grey shading shows genes that are not potential targets.The targets analysed *in vitro* and *in vivo* are shown in red or blue, respectively.

**No.**	**gSELEX peak type**	**Map position (bp**)	**RpoN holo+NtrC**	**RpoN holo**	**NtrC**	**Group**	**Regulon RpoN**	**Regulon NtrC**	**ChIP-chip**	**Operon**	**Left gene**	**D**	**Test protein**	**D**	**Right gene**	**Operon**
1	A	42 372								*caiTABCDE*	*caiT*	<		>	*fixA*	*fixABCX*
2	A	77 346				II-A				***sgrR-sroA-tbpA-thiPQ***	*sgrR*	<		>	*sgrS*	*sgrST-setA*
3	B	257 850									*frsA*	>		>	*crl*	*crl*
4	A	347 864								*prpR*	*prpR*	<		>	*prpB*	*prpBCDE*
5	A	367 650								*mhpR-lacI*	*mhpR*	<		>	*mhpA*	*mhpABCDFE*
6	B	371 336									*mhpC*	>		>	*mhpD*	*mhpDFE*
7	B	418 832									*phoR*	>		>	*brnQ*	*brnQ-proY*
8	B	433 872									*ribD*	>		>	*ribE*	*ribE-nusB-thiL-pgpA*
9	A	443 846								*panE-yajL*	*panE*	<		>	*yajQ*	*yajQ*
10	B	471 846									*mdlB*	>		>	*glnK*	*glnK-amtB*
11	B	547 672									*fdrA*	>		>	*ylbF*	*ylbF-ybcF*
12	B	619 432								*fepC*	*fepC*	<		<	*fepG*	
13	A	655 760								*dcuC*	*dcuC*	<		>	*pagP*	*pagP*
14	B	688 560								*insH*	*insH*	<		<	*lnt*	
15	A	784 656								*ybgS*	*ybgS*	<		>	*aroG*	*aroG*
16	B	847 362								*glnHPQ*	*glnH*	<		<	*dps*	
17	B	874 568									*yliE*	>		>	*yliF*	*yliF*
18	A	882 830								*ybjG*	*ybjG*	<		>	*cmr*	*cmr*
19	B	891 170									*nfsA*	>		>	*rimK*	*rimK-ybjN*
20	B	892 632									*ybjN*	>		>	***potF***	***potFGHI***
21	B	894 130				I-B					*potF*	>		>	*potG*	*potGHI*
22	A	1 073 268								*rutABCDEFG*	*rutA*	<		>	*rutR*	*rutR*
23	B	1 177 842									*lolE*	>		>	*nagK*	*nagK-cobB*
24	B	1 191 232								*purB*	*purB*	<		<	*hflD*	
25	A	1 250 156								*dhaKLM*	*dhaK*	<		>	*dhaR*	*dhaR*
26	B	1 308 556								*kch*	*kch*	<		<	*yciI*	
27	A	1 366 070								*pspF*	*pspF*	<		>	*pspA*	*pspABCDE*
28	B	1 527 534									*yncH*	>		>	*ydcD*	*ydcD*
29	B	1 561 132				II-B				***ddpXABCDF***	*ddpX*	<		<	*dos*	
30	B	1 613 766									*yneJ*	>		>	*yneK*	*yneK*
31	A	1 630 062								*ydfI*	*ydfI*	<		>	*ydfK*	*ydfK*
32	B	1 653 158								*rspAB*	*rspA*	<		<	*ynfA*	
33	B	1 678 972									*ydgI*	>		>	*folM*	*folM*
34	B	1 709 534				II-B					*rsxE*	>		>	*nth*	*nth*
35	A	1 830 436								*astCADBE*	*astC*	<		>	*xthA*	*xthA*
36	B	1 863 654				I-B				*yeaE*	*yeaE*	<		<	*mipA*	
37	B	1 905 652				II-B				***yobF-cspC***	*yobF*	<		<	*yebO*	
38	A	2 036 832								*yedWV*	*yedW*	<		>	*hiuH*	*hiuH*
39	A	2 060 070								*nac*	*nac*	<		>	*asnV*	*asnV*
40	B	2 184 766									*rcnA*	>		>	*rcnB*	*rcnB*
41	B	2 321 470									*atoC*	>		>	*atoD*	*atoDAEB*
42	B	2 360 468								*yfaX-rhmD-yfaVU*	*yfaX*	<		<	*yfaY*	
43	A	2 411 432								*yfbV*	*yfbV*	<		>	*ackA*	*ackA-pta*
44	B	2 425 832								*argT-hisJQMP*	*argT*	<		<	*ubiX*	
45	B	2 429 072								*cvpA-purF-ubiX*	*cvpA*	<		<	*dedD*	
46	A	2 458 968				I-A				*yfcZ*	*yfcZ*	<		>	*fadL*	*fadL*
47	A	2 493 362								*yfdY*	*yfdY*	<		>	*lpxP*	*lpxP*
48	B	2 520 564								*xapR*	*xapR*	<		<	*xapB*	
49	B	2 522 072								*xapB*	*xapB*	<		<	*xapA*	
50	A	2 529 354								*zipA*	*zipA*	<		>	*cysZ*	*cysZ*
51	B	2 531 464									*cysK*	>		>	*ptsH*	*ptsHI-crr*
52	B	2 599 140									*bcp*	>		>	*hyfA*	*hyfABCDEFGHIJR-focB*
53	B	2 689 364								*glmY*	*glmY*	<		<	*purL*	
54	B	2 825 748									*srlB*	>		>	*srlD*	*srlD-gutM-srlR-gutQ*
55	A	2 830 336								*norR*	*norR*	<		>	*norV*	*norVW*
56	B	2 836 270								*hydN-hypF*	*hydN*	<		<	*ascG*	
57	A	2 848 650								*hycABCDEFGHI*	*hycA*	<		>	*hypA*	*hypABCDE-fhlA*
58	B	3 001 538									*xdhB*	>		>	*xdhC*	*xdhC*
59	A	3 004 270								*ygeV*	*ygeV*	<		>	*ygeW*	*ygeW*
60	B	3 043 930								*ygfF*	*ygfF*	<		<	*gcvP*	
61	B	3 370 654								*nanTEK-yhcH*	*nanT*	<		<	*nanA*	
62	B	3 408 032									*prmA*	>		>	*dusB*	*dusB-fis*
63	B	3 417 032									*yhdV*	>		>	*yhdX*	*yhdXYZ*
64	B	3 440 634								*rpsMKD-rpoA-rplQ*	*rpsM*	<		<	*rpmJ*	
65	B	3 446 170								*rplNXE-rpsNH-rplFR-rpsE-rpmD-rplO-secY-rpmJ*	*rplN*	<		<	*rpsQ*	
66	B	3 598 870								*rpoH*	*rpoH*	<		<	*ftsX*	
67	B	3 809 172				II-B				*mutM*	*mutM*	<		<	*rpmG*	
68	A	3 851 352								*istR*	*istR*	<		>	*tisB*	*tisB*
69	B	3 967 058									*rfe*	>		>	*wzzE*	*wzzE-wecBC-rffGHCA-wzxE-rffT-wzyE-rffM*
70	B	3 933 336									*rbsA*	>		>	*rbsC*	*rbsCBKR*
71	B	3 994 336									*yigA*	>		>	*xerC*	*xerC-yigB*
72	B	4 008 248									*pldB*	>		>	*yigL*	*yigL*
73	A	4 056 244								***glnALG***	***glnA***	<		>	*typA*	*typA*
74	A	4 083 972								*fdoGHI-fdhE*	*fdoG*	<		>	*fdhD*	*fdhD*
75	B	4 131 538									*metF*	>		>	*katG*	*katG*
76	A	4 173 336								*coaA*	*coaA*	<		>	*thrU*	*thrU-tyrU-glyT-thrT-tufB*
77	A	4 199 860								*zraP*	*zraP*	<		>	*zraS*	*zraSR*
78	B	4 260 864									*dusA*	>		>	*pspG*	*pspG*
79	B	4 297 530								*fdhF*	*fdhF*	<		<	*mdtP*	
80	B	4 304 842				I-B				*yjcS*	*yjcS*	<		<	*alsK*	
81	B	4 331 330								*basS*	*basS*	<		<	*basR*	
82	B	4 530 150								*sgcXBCQAER*	*sgcX*	<		<	*yjhP*	
	**No. spacers**		**61**	**44**	**30**		**24**	**10**	**24**							
	**No. targets**		**61–80**	**44–61**	**30–41**		**39%**	**33%**	**39%**							

#### RT-qPCR assay *in vivo*

All seven promoter probes containing nine promoters were found to interact with both the RpoN holoenzyme and NtrC by the gel-shift assay *in vitro*. Next, we examined these interactions *in vivo* using an RT-qPCR assay. In addition to the seven probes employed in the gel-shift assay, we added an additional six probes in the *in vivo* assay, including *ntrC* (TF; under the control of the *glnA* promoter), *potI* (putrescine transporter; under the control of the *potF* promoter), *ddpF* (ABC-family transporter; under the control of *ddpX* promoter), *thiQ* (thiamine transporter; under the control of the *sgrR* promoter), *setA* (sugar efflux system; under the control of the *sgrS* promoter) and *cspC* (stress protein; under the control of the *yobF* promoter). All these genes were organized into their corresponding operons under the control of the NtrC regulator; the operons, including these six target genes, are indicated in Table 5.

**Fig. 7.**
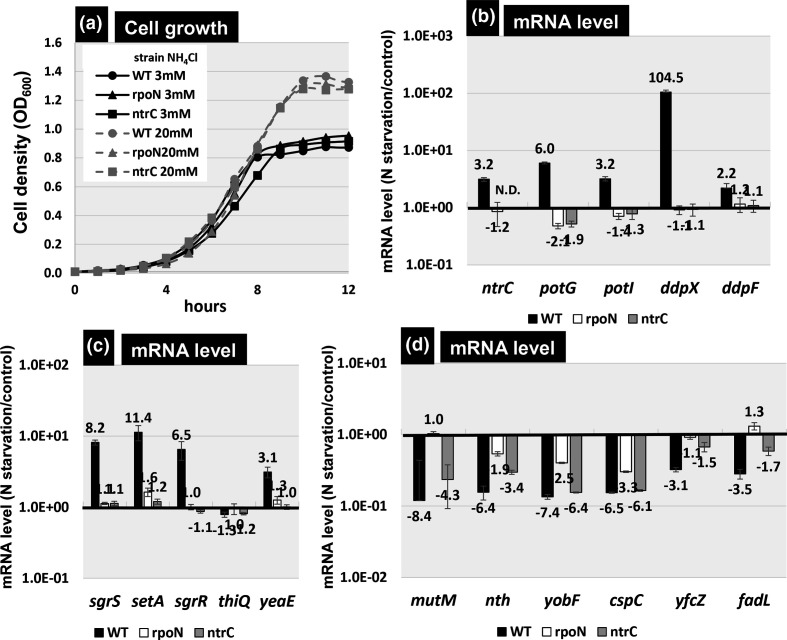
Influence of *rpoN* or *ntrC* on the transcription levels of the newly identified NtrC-dependent RpoN targets. Growth curve of *E. coli* wild-type BW25113, the *rpoN*-deleted mutant JW3169 and the *ntrC*-deleted mutant JW3839 in Gutnick medium supplemented with 0.2% glucose with 3 or 20 mM NH_4_Cl as the sole nitrogen source (**a**). The mRNA levels of the known targets (**b**) and new targets (**c, d**) of the wild-type, *rpoN* mutation and *ntrC* mutation strains under nitrogen depletion. Total RNA was prepared from the wild-type, *rpoN* mutation and *ntrC* mutation strains in the exponential phase (OD_600_ 0.4) and the stationary phase (20 min after growth stopped) in Gutnick medium supplemented with 0.2% glucose and 3 mM NH_4_Cl and, subsequently, it was subjected to RT-qPCR analysis. RT-qPCR was repeated at least three times, and the mean values of the experiments are shown. The *y*-axis represents the relative level of mRNA of each NtrC-dependent RpoN target gene between the nitrogen replete and deplete conditions, with the ratio of 16S rRNA used as an internal control in each strain. nd indicates it was not detected as below the detection limit.

Intracellular levels of RpoN and NtrC are activated in response to nitrogen depletion [6, 47]. Gutnick minimal medium is widely used as a nitrogen source control medium [21, 34]. We observed the growth of the wild-type *E. coli* K-12 BW25113 strain and its *rpoN* and *ntrC* mutation strains. In the nitrogen-rich medium (supplemented with 20 mM NH_4_Cl), the cell density of all three strains reached OD_600_ 1.3 (Fig. 7a) at 10 h after inoculation. However, in the nitrogen-limited medium (supplemented with 3 mM NH_4_Cl), the cell growth of all these strains was delayed at an OD_600_ of 0.9, 8 h after inoculation. Based on this result, each of the wild-type, *rpoN*-deleted and *ntrC*-deleted strains were inoculated in nitrogen-limited Gutnick medium, and total RNA was prepared from the nitrogen replete phase (OD_600_=0.4) or depleted phase (OD_600_=0.9). The mRNA level was then measured for 17 representative target genes.

At present, the three representative promoters (*ntrC*, *potG* and *ddpX*) are known to be recognized by the RpoN sigma factor and activated by NtrC [12]. After repeated RT-qPCR analysis, the mRNA levels of *ntrC* were found to increase under nitrogen-depleted conditions (Fig. 7b, *ntrC* slot), in agreement with the proposed induction of NtrC by nitrogen depletion [12]. As expected, *ntrC* induction was not observed in the *rpoN*-defective mutant, and negligible levels of *ntrC* mRNA were detected in the *ntrC*-defective mutant. The *potG* gene is a member of *the potGHI* operon for the putrescine ABC transporter, and the *ddpX* gene forms the *ddpXABCDF* operon for d-alanyl-d-alanine dipeptidase and d-Ala-d-Ala transporter. The *potG* and *ddpX* promoters are known regulatory targets of the NtrC-dependent RpoN holoenzyme. Both promoters have been confirmed as regulatory targets of the NtrC-dependent RpoN holoenzyme, via gSELEX screening and the gel-shift assay. To measure the expression of these two operons, the mRNA levels were determined for the first (*potG* and *ddpX*) and last (*potI* and *ddpF*) genes of each operon. The levels of *potG* and *potI* mRNA increased by 6.0- and 3.2-fold, respectively, while the *ddpX* and *ddpF* mRNA levels increased by 104.5- and 2.2-fold, respectively (Fig. 7b). Thus, we concluded that the *potGHI* and *ddpXABCDF* operons were markedly activated in the presence of both RpoN and NtrC.

Next, we examined the expression of newly identified target genes or operons of the NtrC-dependent RpoN holoenzyme (Fig. 7c). The *sgrST-setA* operon produces *sgrS* sRNA for the translation inhibition of *ptsG* mRNA, the SgrT inhibitor of glucose transporter PtsG and the sugar exporter SetA. mRNA for the *sgrST-setA* operon increased 8.2-fold, when detected with *sgrS*, and 11.4-fold, when detected with *setA*, in the wild-type *E. coli* K-12 strain under nitrogen depletion. We also analysed the expression of the *sgrR-sroA-thiBPQ* operon, which is located divergently from the *sgrST-setA* operon. SgrR is a transcription activator for the *sgrST-setA* operon, and *sroA* sRNA regulates the translation of ThiBPQ, a thiamine transporter. The transcription unit of *sgrR-sroA-thiBPQ* was inferred automatically computationally without experimental confirmation [50]. Under nitrogen-depleted conditions, the mRNA levels for the first gene *sgrR* increased 6.5-fold (Fig. 7c), while the level of *thiQ*, the last gene of this predicted operon, did not increase, implying that *thiQ* is not organized in this operon. The mRNA levels of the divergently organized *sgrST-setA* operon and the divergently transcribed *sgrR* gene did not increase in the *rpoN* and *ntrC* mutants. Thus, we concluded that the *sgrST-setA* operon and the divergently transcribed *sgrR* gene encoding the regulator of the *sgrST-setA* operon are under the control of the NtrC-dependent RpoN holoenzyme. The *yeaE* gene was identified as another novel target of the NtrC-dependent RpoN holoenzyme. YeaE (methylglyoxal reductase) converts methylglyoxal to hydroxyacetone. *yeaE* mRNA increased 3.1-fold under nitrogen depletion (Fig. 7c), but not in *rpoN* and *ntrC* mutants, indicating that the *yeaE* promoter was regulated by the NtrC-dependent RpoN holoenzyme.

In contrast, mRNA levels of the other seven newly identified target genes decreased in wild-type *E. coli* K-12 under nitrogen-depleted conditions (Fig. 7d). Both *mutM* mRNA and *nth* mRNA expression decreased, by 8.4-fold and 6.4-fold, respectively. MutM (DNA-formamidopyrimidine glycosylase) is a DNA glycosylase for redox-damaged purine nucleotides, whereas Nth (endonuclease III) is a DNA glycosylase and apurinic/apyrimidinic lyase for the repair of DNA damage. These proteins appear to be involved in the modulation of DNA structure and function, which is not necessary under nitrogen-depleted conditions. In the absence of RpoN sigma, however, this reduction in the mRNA levels of *mutM* and *nth* was not observed in the *rpoN*-deleted mutant (Fig. 7d). This finding supports the repressor role of the RpoN holoenzyme, irrespective of the presence or absence of NtrC.

CspC is a member of the cold shock proteins [51] that has the ability to bind RNA and ssDNA for the modulation of DNA/RNA functions. For instance, CspC acts as a transcription anti-terminator. The *cspC* gene forms an operon with the currently uncharacterized *yobF* gene. Levels of *cspC* and *yobF* mRNA were found to decrease 6.5- and 7.4-fold, respectively, under nitrogen-depleted conditions. Reduction in the levels of *yobF* and *cspC* mRNA decreased in the *rpoN* mutant, while a negligible effect was observed in the *ntrC* mutant (Fig. 7d). The *fadL* (fatty acid uptake outer membrane channel) and *yfcZ* genes form a divergent transcription unit. The levels of *fadL* and *yfc*Z mRNA decreased 3.5- and 3.1-fold, respectively (Fig. 7d), supporting the repressor role of the RpoN holoenzyme. These results suggest that the RpoN holoenzyme functions as a repressor for a set of genes.

To confirm the repressive function of NtrC-dependent RpoN holoenzyme binding, we further carried out an *in vitro* competition assay for four targets between RpoN holoenzyme against RNAP containing the major sigma factor RpoD in the presence or absence of NtrC. At first, each of these test probes was mixed with purified RpoN holoenzyme with or without NtrC or RpoD holoenzyme, and the probe–test protein mixtures were then subjected to PAGE. The complexes of RpoN holoenzyme–probe, RpoN holoenzyme–NtrC–probe and RpoD holoenzyme–probe were observed as shift bands (Fig. S1a). Next, RpoD holoenzyme was added into the mixture in which the target probe formed complex with RpoN holoenzyme under the presence or absence of NtrC, and then subjected to PAGE. The probe pattern was similar to that in the presence of RpoN holoenzyme with or without NtrC (Fig. S1a). To confirm the binding of each RNAP holoenzyme, we then performed Western blot analysis using antibodies against RpoN and RpoD. Using the RpoN antibody, the signal was observed for RpoN holoenzyme and the intensity was higher in the presence of NtrC, which suggests the binding of RpoN holoenzyme to the target promoter was supported by NtrC (Fig. S1b). In contrast, the signal of RpoD was detected in RpoD holoenzyme alone, but the intensity became low under the presence of RpoN holoenzyme, and almost disappeared with addition of NtrC. These results suggest that the binding of NtrC supported RpoN holoenzyme competes against RpoD holoenzyme bindings due to their ability to repress these promoters. Thus, we propose naming the group of promoters recognized by the RpoN holoenzyme alone for the repression of the target genes as the group of repressive promoters. The RpoN holoenzyme acts as a repressor for the transcription of a group of target genes.

### Physiological roles of the RpoN promoters: a class of repressive promoters

We previously identified a set of constitutive promoters recognized by RNAP alone containing each of the major sigma factors of RpoD and the minor sigma factors (stationary-phase sigma RpoS, heat-shock sigma RpoH, flagella-chemotaxis sigma RpoF and extra-cytoplasmid sigma RpoE) using the gSELEX system [26, 27]. Constitutive promoters are recognized and transcribed by each of these RNAP holoenzymes alone in the absence of supporting regulatory proteins. In the case of RpoN, however, the RNAP RpoN holoenzyme recognizes and binds to a set of promoters, detected here using gSELEX screening. For transcription initiation, TFs with an enhancing role have been proposed, including those in the NtrC family (eight species in *E. coli *) or TyrR family (four species) [6, 16]. The experimental confirmation of this enhancer role has only been obtained with a few enhancers, including NtrC [8] and PspF [52]. In this study, we identified the whole set of binding sites for the RpoN holoenzyme in the presence and absence of the enhancer NtrC.

A total of 71 binding sites were identified by the RpoN holoenzyme alone, of which 23 (32 %) are listed in RegulonDB database (Table S2). In the presence of NtrC, a total of 108 binding sites were identified by the RpoN holoenzyme in the presence of NtrC, of which 28 (26 %) binding sites are listed in RegulonDB [50]. In RegulonDB, a total of 101 targets were listed as RpoN-dependent promoters; however, only seven targets (*acrD*, *actP*, *aslB*, *astC*, *chaC*, *ddpX* and *emrD*) have been experimentally confirmed. All these targets were identified using *in vitro* gSELEX screening, indicating the degree of reliability of gSELEX screening. The majority of the other targets listed in RegulonDB were predicted by computational approaches relying on consensus sequences obtained from a small number of known promoters.

RpoN regulates not only genes involved in nitrogen metabolism but also other cellular functions, including metabolic pathways, dependent on different enhancers, such as formate catabolism (TyrR family; FhlA-dependent), acetoacetate catabolism (NtrC family; AtoC-dependent), propionate catabolism (TyrR family; PrpR-dependent), phage shock response (NtrC family; PspF-dependent) and zinc response (NtrC family; ZraR-dependent) [6]. We further identified a novel set of genes under the control of NtrC-dependent RpoN holoenzyme involved in carbon source metabolism, such as the *sgrST-setA* operon (inhibition of PtsG glucose transporter and sugar efflux pump) and the *sgrR* gene (activation of the *sgrST-setA* operon) [53, 54], both activated by NtrC. The activation of glucose transporter inhibitors and sugar efflux pumps, together with the repression of a long-chain fatty acid transporter, may lead to a carbon/nitrogen imbalance by decreasing the carbon source influx and increasing the carbon source efflux. Upon accumulation, 2-oxoglutamate, the key metabolite of the intersection between carbon and nitrogen metabolism, binds to GlnB (nitrogen regulatory protein PII) and activates NtrB sensor kinase, which phosphorylates NtrC, thereby activating a number of NtrC-dependent genes. The expression of the *ntrBC* (*glnLG*) operon is auto-regulated by NtrB-phosphorylated NtrC, depending on nitrogen availability.

Several promoters recognized by the RpoN holoenzyme alone were identified as repressive promoters. The occupation of repressive promoters by the RpoN holoenzyme was predicted to inhibit the expression of the genes located downstream. In the absence of enhancers under steady-state stressless conditions, the repressive promoters must be occupied by the RNAP RpoN holoenzyme, to prevent transcription by nearby promoters that are recognized by the holoenzymes containing sigma factors other than RpoN. In fact, the binding of the NtrC-enhanced RpoN holoenzyme to the targets interfered with the binding to the promoter of the RpoD holoenzyme (Fig. S1). Promoter sequences recognized by other sigma factors overlapped with the newly identified repressive promoters, such as the RpoD promoter on the *yobF-cspC* promoter, and RpoH and RpoD promoters on the *mutM* promoter (listed in Regulon DB). Our observations of the repressive promoters are in good agreement with the proposed model of the repressor role for RpoN in transcription of some genes [18]. In *E. coli* K-12 cells grown in rich media (e.g. LB broth), three species of the sigma subunit are present: (i) RpoD, the most abundant and responsible for the transcription of growth-related genes; (ii) RpoN, the second most abundant; (iii) RpoF, responsible for the transcription of flagella-chemotaxis gene [36, 37]. To date, the mechanism underlying the presence of high levels of RpoN in growing *E. coli* cells has yet to be elucidated, even though nitrogen is not limiting. One possibility is that the RpoN holoenzyme exerts a repressor role to prevent the transcription of a certain group of genes by the most abundant RpoD holoenzyme. The presence of the RpoN holoenzyme on repressive promoters allows transcription to initiate quickly once enhancer proteins are induced under the corresponding stress conditions, such as NtrC activation under nitrogen depletion.

In this study, we identified the whole set of binding sites of RpoN holoenzyme, the enhancer NtrC and NtrC-dependent RpoN holoenzyme using *in vitro* gSELEX-chip screening in the absence of other regulators. The identification of a set of promoters recognized by the RpoN holoenzyme in the presence and absence of NtrC enhancer, including the repressive promoters, provides insight into the regulation of the bacterial genome.

## Supplementary Data

Supplementary material 1
