# Supplementary material for: Whole set of constitutive promoters for RpoN sigma factor and the regulatory role of its enhancer protein NtrC in Escherichia coli K-12
Source: Microb Genom. 2021 Nov 17;7(11):000653. doi: 10.1099/mgen.0.000653 (PMC8743547; doi:10.1099/mgen.0.000653)
Supplement: Supplementary material 1 [file mgen-7-0653-s001.pdf]

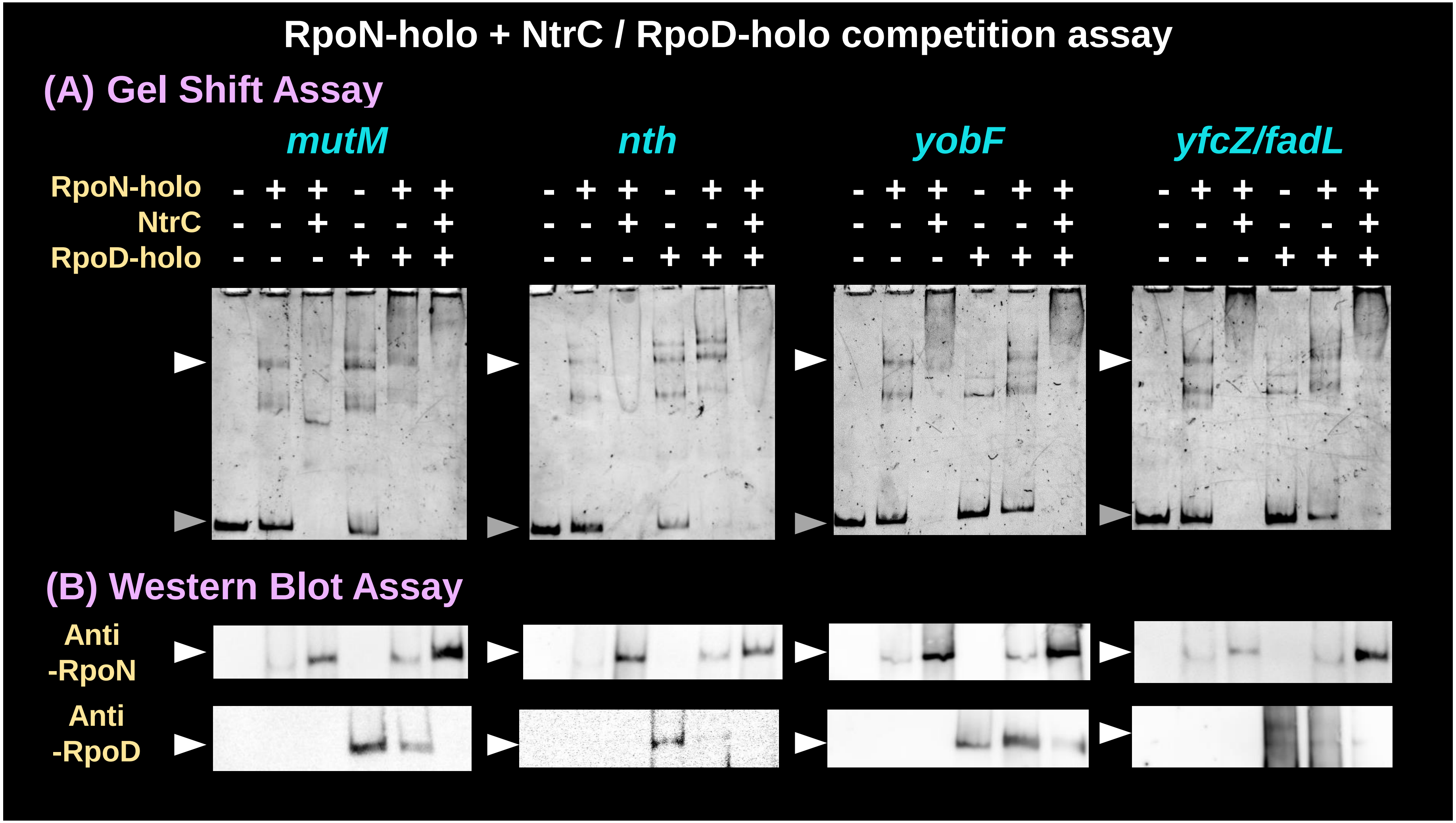

Figure S1. Effect of NtrC enhanced RpoN holoenzyme binding on the repressive promoters against RpoD holoenzyme binding. The RpoN target promoter fragments (0.2 μM) were mixed with the RpoN holoenzyme (0.4 μM, lane 2, 3, 5, and 6), NtrC (20 μM, lane 3, and 6), and RpoD holoenzyme (0.5 μM, lane 4, 5, and 6) (lane 5 and 6, RpoN holoenzyme and NtrC were added and incubated for extra 15 min before addition of RpoD holoenzyme). For NtrC activation, 25 mM of each acetyl-phosphate and ATP were added in all conditions. After incubation at 37° C for 30 min, the reaction mixture was subjected to 3.5% PAGE. The PAGE gel was stained by Gel Red for detection of probe DNA (A). Gray triangles indicate the free probe and white triangles indicate the test RNAP-DNA probe complex. The PAGE gel was subjected to western blot analysis and detected by using anti-RpoN or anti-RpoD (B). White triangles indicate the same position on the blot membrane as white triangles on the panel (A).

Table S1. Primers used in this study.

(A) For gel shift assay

name	sequence
potG-F	CCTATCAGTTCCTGAATTACC
potG-R	GTAGGATTTGGTCAGGTTG
ddpX-F	TATTTCTTTTCCCGCCCCCTAC
ddpX-R	GCGCAGGCGTATTTCAATTC
yfcZ/fadL-F	CCGGAATTCAAGAAAATTCGTTTAACTGAAA
yfcZ/fadL-R	CGCGGATCCCGTTGATTTCCTCTGTATGT
yeaE-F	ACCAGAACGAATACTATTATGG
yeaE-R	CCATATACCATGTTCCCTG
yjcS-F	CTCATCTTCTGACTTTAATGG
yjcS-R	CGAGAGTTATTCATATAAATCTCC
sgrR/sgrS-F	CAAGTCAACTTTCAGAATTG
sgrR/sgrS-R	TTGTGTCCTGCGATTTAC
mutM-F	TGCGCGTATCTGCTAAAGGT
mutM-R	ACGAGATGCGGTTCTATGCC
yobF-F	GGTATTATTTATTAGCGAAACG
yobF-R	AAATGCCACACATAAACAG
nth-F	GTCACTAACTGTATCGTTGTG
nth-R	ATGAGGATTGTTCTCACG
lacUV5-F	CAGCTGGCACGACAGGTTTC
lacUV5-R	AGCTGTTTCCTGTGTGAAATTG

(B) For RT-qPCR analysis

ntrC-F	TAAACAGCGCCATCCAATGC
ntrC-R	AAACGCCCCTTGTTGATAGG
potG-F	TGCTTGATGGCGTCGATTTG
potG-R	TCAGGCCAAAAGCGATGTTC
potI-F	GGGCTTGTCGCTGTTGTTAT
potI-R	ATGAGGATTGTTCTCACG
ddpX-F	TTATCAGCAAGCGCGTTGTC
ddpX-R	AACATCGCCTGTGCTTGTTG
ddpF-F	ATTTCCCGGCCCGTAAAAAC
ddpF-R	ACGATCCCTAAGGTTTCACCAC
yeaE-F	TATTGTTGGCGTGGGTGATC
yeaE-R	ACAGCCGCATTTTGTTGGAC
sgrR-F	GCAAGTCAGGGACTGGGTAA

sgrR-R	AAAGGTTAGGGTGAGGCTTTCC
setA-F	AGCGAGTTAGGATTGCCAGA
setA-R	ATTCGCCGCTTACCATAACG
mutM-F	TGCCAGCGAATCACTGTTTG
mutM-R	TCAATCGAACGCAGCAACAC
nth-F	CGCCTTTTGAATTGCTGATT
nth-R	AGGTTTTCACCCCTTCAACG
yjcS-F	TCGTCCGCAAAAACCGATTG
yjcS-R	TTTTCGCTGATGGCTTCGTC
yobF-F	GTCCTGAGTAAACACGTTGACG
yobF-R	ACTGACACATTACTGCATGAGG
cspC-F	TGGCAGCAAAGATGTGTTCG
cspC-R	TTCTGGCCGTCCTGAATTTC
yfcZ-F	TGACTGAAAAAGCCCGTAGC
yfcZ-R	TTCAGCTTCGCAGGCAAAAG
fadL-F	GCCTGAAATGTGGGAAGTGT
fadL-R	CGGTACGGAAGGTCCAGTTA
rrsA-F	TGCATCTGATACTGGCAAGC
rrsA-R	TACGCATTTCACCGCTACAC

Table S2

RpoN holoenzyme-binding sites on the *E. coli* K-12 genome

No	gSELEX peak type	Map position (bp)	RpoN holo	Regul on DB	ChIP-chip	Left Gene Function	Operon	Left Gene	D	RpoN holo site	D	Right Gene	Operon	Right Gene Function	RpoN promoter motif (ntGGcacnnnnnttGCn)
1	D	46866	32%			predicted 4Fe-4S ferredoxin-type protein		*fixX*	>	*yaaU*	>	*kefF*	*kefFC*	flavoprotein subunit for the KefC potassium efflux system	TTGGCACCATTGTTTCG
2	D	261272	56%			gamma-glutamate kinase		*proB*	>	*proA*	>	*thrW*	*thrW*	Thr tRNA	ACGGCACAGTTTATGCA
3	D	332440	30%			choline transporter of high affinity		*betT*	>	*yahA*	<	*yahB*		predicted DNA-bindng transcriptional regulator	TTGGTACGGGTTATGCG
4	A	347864	47%			DNA-binding transcriptional activator	*prpR*	*prpR*	<		>	*prpB*	*prpBCDE*	2-methylisocitrate lyase	GTGGCACACCCCTTGCT
5	D	415552	40%			exonuclease, dsDNA, ATP-dependent	*sbcC*	*sbcC*	<	*sbcD*	>	*phoB*	*phoBR*	DNA-binding response regulator in two-component regulatory system with PhoR (or CreC)	CCGGCACAAAACTTTCC
6	B	471846	61%			fused predicted multidrug transporter subunits of ABC superfamily: ATP-binding components		*mdlB*	>		>	*glnK*	*glnK-amtB*	nitrogen assimilation regulatory protein for GlnL, GlnE, and AmtB	CTGGCACACCGCTTGCA
7	D	503730	45%			predicted transporter with NAD(P)-binding Rossmann-fold domain	*ybaL*	*ybaL*	<	*fsr*	>	*ushA*	*ushA*	bifunctional UDP-sugar hydrolase/5'-nucleotidase	ATGCCATGGTCGTTGCC
8	D	601842	31%			copper/silver efflux system, membrane component		*cusA*	>	*pheP*	<	*ybdG*		predicted mechanosensitive channel	TTGGAATGCTTTTTTCC
9	B	619432	39%			iron-enterobactin transporter subunit	*fepC*	*fepC*	<		<	*fepG*		iron-enterobactin transporter subunit	TTGGCCCGATAATTGCC
10	A	655760	35%			anaerobic C4-dicarboxylate transport	*dcuC*	*dcuC*	<		>	*pagP*	*pagP*	palmitoyl transferase for Lipid A	TTGGTAAAGTTTTTGCT
11	B	688560	55%			IS5 transposase and trans-activator	*insH*	*insH*	<		<	*lnt*		apolipoprotein N-acyltransferase	TTGGCACATCTATTGCT
12	D	702662	56%			N-acetylglucosamine-6-phosphate deacetylase	*nagAC-umpH*	*nagA*	<	*nagB*	>	*nagE*	*nagE*	fused N-acetyl glucosamine specific PTS enzyme: IIC, IIB , and IIA components	CTGGCCTGCTTTATGCA
13	A	784656	32%			conserved protein	*ybgS*	*ybgS*	<		>	*aroG*	*aroG*	3-deoxy-D-arabino-heptulosonate-7-phosphate synthase, phenylalanine repressible	CTGGCGTTTTTCTTGCT
14	D	797452	38%			molybdate transporter subunit		*modC*	>	*ybhA*	>	*pgl*	*pgl*	6-phosphogluconolactonase	CCAGCACGGTTTTTGCA
15	B	847362	74%			glutamine transporter subunit	*glnHPQ*	*glnH*	<		<	*dps*		Fe-binding and storage protein	CTGGCACGATTTTTTCA
16	D	848670	47%			Fe-binding and storage protein	*dps*	*dps*	<	*rhtA*	>	*ompX*	*ompX*	outer membrane protein	TTGGCACGAAAATTAAC
17	B	874568	32%			conserved inner membrane protein		*yliE*	>		>	*yliF*	*yliF*	predicted diguanylate cyclase	GTAGCGTGGCAATTGAT
18	A	882830	57%			undecaprenyl pyrophosphate phosphatase	*ybjG*	*ybjG*	<		>	*cmr*	*cmr*	multidrug efflux system protein	TTGGCGAAGAAATTGCA
19	D	884934	32%			predicted protein	*ybjH*	*ybjH*	<	*ybjI*	<	*ybjJ*		predicted transporter	TAGGCACTATTTTTTCC
20	B	891170	44%			nitroreductase A, NADPH-dependent, FMN-dependent		*nfsA*	>		>	*rimK*	*rimK-ybjN*	ribosomal protein S6 modification protein	TTGGCACCGCCATTACC
21	B	892632	100%			predicted oxidoreductase		*ybjN*	>		>	*potF*	*potFGHI*	putrescine transporter subunit: periplasmic-binding component of ABC superfamily	TTTGCATGGTTCGTGCG
22	D	968834	32%			lipid A 4'kinase		*lpxK*	>	*ycaQ*	>	*ycaR*	*ycaR-kdsB*	conserved protein	ATTGCACAGTTAATTCA
23	A	1073268	71%			predicted monooxygenase	*rutABCDEFG*	*rutA*	<		>	*rutR*	*rutR*	predicted DNA-binding transcriptional regulator	CTGGCATCCGCTTTGCA
24	D	1087762	50%			predicted glycosyl transferase	*pgaCD*	*pgaC*	<	*pgaB*	<	*pgaA*		predicted outer membrane protein	TTGGCATGTTATATGAA
25	D	1138870	36%			muramidase		*flgJ*	>	*flgK*	>	*flgL*	*flgL*	flagellar hook-filament junction protein	GTGCCACGGTAACTGAT
26	B	1191232	41%			adenylosuccinate lyase	*purB*	*purB*	<		<	*hflD*		predicted lysogenization regulator	GCTGCGCGGGATTTTCA
27	B	1308556	65%			voltage-gated potassium channel	*kch*	*kch*	<		<	*yciI*		predicted enzyme	AGGGCACGGTTTTTGCA
28	A	1366070	68%			DNA-binding transcriptional activator	*pspF*	*pspF*	<		>	*pspA*	*pspABCDE*	regulatory protein for phage-shock-protein operon	TTGGCACGCAAATTGTA
29	B	1527534	30%			predicted protein		*yncH*	>		>	*ydcD*	*ydcD*	predicted protein	GTGGACTAATTTATGCC
30	D	1553332	33%			ethanol-active dehydrogenase/acetaldehyde-active reductase	*adhP*	*adhP*	>	*maeA*	<	*sra*		30S ribosomal subunit protein S22	GCGGCGTGTTTATCTCT
31	D	1644062	36%			Qin prophage; bifunctional antitoxin of the RelE-RelB toxin-antitoxin system/ transcriptional repressor	*relBE-hokD*	*relB*	<	*ydfV*	>	*flxA*	*flxA*	Qin prophage; predicted protein	GTAACACGTTTTATGCA
32	D	1790536	38%			conserved protein	*ydiV*	*ydiV*	<	*nlpC*	<	*btuD*		vitamin B12 transporter subunit : ATP-binding component of ABC superfamily	TTGGCACGCAAATTGAT
33	A	1830436	50%			succinylornithine transaminase, PLP-dependent	*astCADBE*	*astC*	<		>	*xthA*	*xthA*	exonuclease III	ATGGCGCAGTAATTTCC
34	D	1838132	30%			predicted transporter subunit: ATP-binding component of ABC superfamily		*ynjD*	>	*ynjE*	<	*ynjF*		predicted phosphatidyl transferase, inner membrane protein	GTGGCGCAGATTATGCT
35	D	1869670	64%			conserved protein		*yeaH*	>	*yeaI*	>	*yeaJ*	*yeaJ*	predicted diguanylate cyclase	TTGGCACGGTTTTTTCA
36	A	2036832	31%			predicted DNA-binding response regulator in two-component system with YedV	*yedWV*	*yedW*	<		>	*hiuH*	*hiuH*	hydroxyisourate hydrolase / transthyretin-related protein	ATGGCATATTATTTTCA
37	A	2060070	52%			DNA-binding transcriptional dual regulator of nitrogen assimilation	*nac*	*nac*	<		>	*asnV*	*asnV*	Asn tRNA	CTGGCAAGCATCTTGCA
38	D	2317672	39%			DNA-binding response regulator in two-component regulatory system with RcsC and YojN		*rcsB*	>	*rcsC*	>	*atoS*	*atoSC*	sensory histidine kinase in two-component regulatory system with AtoC	TTGGCGTGGTTTATGCG
39	D	2318844	54%			hybrid sensory kinase in two-component regulatory system with RcsB and YojN	*rcsC*	*rcsC*	<	*atoS*	>	*atoC*	*atoC*	fused response regulator of ato operon, in two-component system with AtoS: response regulator/sigma54 interaction protein	CTGGCACAAAATATTCC
40	B	2321470	48%			fused response regulator of ato operon, in two-component system with AtoS: response regulator/sigma54 interaction protein		*atoC*	>		>	*atoD*	*atoDAEB*	acetyl-CoA:acetoacetyl-CoA transferase, alpha subunit	CTGGCACTCCCCTTGCT
41	A	2411432	31%			conserved inner membrane protein	*yfbV*	*yfbV*	<		>	*ackA*	*ackA-pta*	acetate kinase A and propionate kinase 2	TTGCCATCATCGATGCA
42	B	2425832	61%			lysine/arginine/ornithine transporter subunit	*argT-hisJQMP*	*argT*	<		<	*ubiX*		3-octaprenyl-4-hydroxybenzoate carboxy-lyase	ATGGCATAAGACCTGCA
43	B	2429072	31%			membrane protein required for colicin V production	*cvpA-purF-ubiX*	*cvpA*	<		<	*dedD*		conserved protein	TCGGTGCAGCTTTTTCT
44	A	2493362	54%			predicted inner membrane protein	*yfdY*	*yfdY*	<		>	*lpxP*	*lpxP*	palmitoleoyl-acyl carrier protein (ACP)-dependent acyltransferase	ACAGCATGTTAAATGCA
45	B	2520564	32%			DNA-binding transcriptional activator	*xapR*	*xapR*	<		<	*xapB*		xanthosine transporter	ATGCCATGATAATTTAA
46	B	2531464	50%			cysteine synthase A, O-acetylserine sulfhydrolase A subunit		*cysK*	>		>	*ptsH*	*ptsHI-crr*	phosphohistidinoprotein-hexose phosphotransferase component of PTS system (Hpr)	CTGGCATTACTGTTGCA
47	D	2584448	30%			fused predicted oxidoreductase: FeS binding subunit/NAD/FAD-binding subunit	*aegA*	*aegA*	<	*narQ*	>	*acrD*	*acrD*	aminoglycoside/multidrug efflux system	ACGGTACAATTTATGCA
48	B	2599140	92%			thiol peroxidase, thioredoxin-dependent		*bcp*	>		>	*hyfA*	*hyfABCDEFGHIJ R-focB*	hydrogenase 4, 4Fe-4S subunit	ATGGCATCCTTTATGCA
49	B	2689364	48%			ncRNA	*glmY*	*glmY*	<		<	*purL*		phosphoribosylformyl-glycineamide synthetase	TTGGCACAGTTACTGCA
50	B	2825748	31%			glucitol/sorbitol-specific enzyme IIA component of PTS		*srlB*	>		>	*srlD*	*srlD-gutM-srlR-gutQ*	sorbitol-6-phosphate dehydrogenase	GTGCCACATTTGCGGCT
51	B	2836270	35%			formate dehydrogenase-H, [4Fe-4S] ferredoxin subunit	*hydN-hypF*	*hydN*	<		<	*ascG*		DNA-binding transcriptional repressor	CTGGCATGATTTGTGAA
52	D	2846068	39%			hydrogenase 3, membrane subunit	*hycDEFGHI*	*hycD*	<	*hycC*	<	*hycB*		hydrogenase 3, Fe-S subunit	GTGGCGCGTTTGTTGCC
53	A	2848650	40%			regulator of the transcriptional regulator FhlA	*hycABCDEFGHI*	*hycA*	<		>	*hypA*	*hypABCDE-fhlA*	protein involved in nickel insertion into hydrogenases 3	CTGGCACAATTATTGCT
54	A	3004270	53%			predicted DNA-binding transcriptional regulator	*ygeV*	*ygeV*	<		>	*ygeW*	*ygeW*	conserved protein	CTGGCACACTTATTGTT
55	B	3043930	39%			predicted NAD(P)-binding oxidoreductase with NAD(P)-binding Rossmann-fold domain	*ygfF*	*ygfF*	<		<	*gcvP*		glycine decarboxylase, PLP-dependent, subunit (protein P) of glycine cleavage complex	CTGGCGCGAATATTTAC
56	D	3318732	30%			argininosuccinate synthetase		*argG*	>	*yhbX*	<	*leuU*		Leu tRNA	TTGCCGCATTTATCGCA
57	D	3330334	31%			GTPase involved in cell partioning and DNA repair	*obgE*	*obgE*	<	*yhbE*	<	*rpmA*		50S ribosomal subunit protein L27	CTGGCAACCATCATGCC
58	B	3417032	40%			predicted outer membrane protein		*yhdV*	>		>	*yhdX*	*yhdXYZ*	predicted amino-acid transporter subunit	CTGGCACTACTTTTGCT
59	B	3440634	32%			30S ribosomal subunit protein S13	*rpsMKD-rpoA-rpl*[illegible]	*rpsM*	<		<	*rpmJ*		50S ribosomal subunit protein L36	TTGGCGCTGTTTATGCT
60	D	3565536	56%			glycogen phosphorylase	*glgP*	*glgP*	<	*glgA*	<	*glgC*		glucose-1-phosphate adenylyltransferase	AAGGCATGTTTTATGCA
61	B	3598870	59%			RNA polymerase, sigma 32 (sigma H) factor	*rpoH*	*rpoH*	<		<	*ftsX*		predicted transporter subunit: membrane component of ABC superfamily	CTGGCACAGTTGTTGCT
62	D	3803540	42%			lipopolysaccharide core biosynthesis protein	*rfaS*	*rfaS*	<	*rfaP*	<	*rfaG*		glucosyltransferase I	ATGGTGCGTAAAATGCA
63	A	3851352	59%			ncRNA	*istR*	*istR*	<		>	*tisB*	*tisB*	lexA-regulated toxic peptide	TCGGCACGAATTTTGAC
64	D	3937930	32%			DNA-binding transcriptional repressor of ribose metabolism		*rbsR*	>	*hsrA*	>	*yieP*		predicted transcriptional regulator	TTGGCGTGCGTCGTGCA
65	B	3967058	52%			UDP-GlcNAc:undecaprenylphosphate GlcNAc-1-phosphate transferase		*rfe*	>		>	*wzzE*	*wzzE-wecBC-rffGHCA-wzxE-rffT-wzyE-rffM*	Entobacterial Common Antigen (ECA) polysaccharide chain length modulation protein	CAGGCACGCTTAATGCA
66	A	4056244	47%			glutamine synthetase	*glnALG*	*glnA*	<		>	*typA*	*typA*	GTP-binding protein	TTGGCACAGATTTCGCT
67	A	4083972	43%			formate dehydrogenase-O, large subunit	*fdoGHI-fdhE*	*fdoG*	<		>	*fdhD*	*fdhD*	formate dehydrogenase formation protein	GCGGCGTGATGATTTAC
68	B	4131538	31%			5,10-methylenetetrahydrofolate reductase		*metF*	>		>	*katG*	*katG*	catalase/hydroperoxidase HPI(I)	TTGGCGCGAATATTGCC
69	A	4199860	60%			Zn-binding periplasmic protein	*zraP*	*zraP*	<		>	*zraS*	*zraSR*	sensory histidine kinase in two-component regulatory system with ZraR	ATGGCATGATTTCTGCT
70	B	4260864	54%			tRNA-dihydrouridine synthase A		*dusA*	>		>	*pspG*	*pspG*	phage shock protein G	TCGGCACTTTTGGTGCT

71	B	4297530	50%			formate dehydrogenase-H, selenopolypeptide subunit	*fdhF*	*fdhF*	<		<	*mdtP*		predicted outer membrane factor of efflux pump	GTGGCATAAAAGATGCA
	A, 17	Cut-off	> 30%					Spacer = 44							
	B, 27	Total	71					ORF = 27							
	C, 0														
	D, 27														

Table S3

NtrC-binding sites on the *E. coli* K-12 genome

No	gSELEX peak type	Map position (bp)	gSELEX NtrC	Regulon DB	Left Gene Function	Operon	Left Gene	D	NtrC-binding site	D	Right Gene	Operon	Right Gene Function	NtrC box (TGCACCAnnnTGGTGCA)
1	D	37572	54%		crotonobetainyl CoA hydratase	caiDE	caiD	<	caiC	<	caiB		crotonobetainyl CoA:carnitine CoA transferase	GGCGCGATTATGGTGCC
2	D	57850	60%		exported protein required for envelope biosynthesis and integrity	lptD-surA-pdxA-rsmA-apaGH	lptD	<	djlA	<	rluA		pseudouridine synthase for 23S rRNA (position 746) and tRNAphe(position 32)	TGAATCTTCATGGCGCT
3	D	327068	45%		choline dehydrogenase, a flavoprotein	betA	betA	<	betB	<	betI		DNA-binding transcriptional repressor	TTTGCCATCATGGCGAT
4	A	367650	88%		DNA-binding transcriptional activator, 3HPP-binding	mhpR-lacI	mhpR	<		>	mhpA	mhpABCDFE	3-(3-hydroxyphenyl)propionate hydroxylase	TTCGCTTAATTGCCGCA
5	B	371336	71%		2-hydroxy-6-ketonona-2,4-dienedioic acid hydrolase		mhpC	>		>	mhpD	mhpDFE	2-keto-4-pentenoate hydratase	CGCAACGTCATGTTGCA
6	D	427046	50%		SecYEG protein translocase auxillary subunit		yajC	>	secD	>	secF	secF	SecYEG protein translocase auxillary subunit	AGCGCCTTCATGGTGCC
7	B	433872	43%		fused diaminohydroxyphosphoribosylaminopyrimidine deaminase and 5-amino-6-(5-phosphoribosylamino) uracil reductase		ribD	>		>	ribE	ribE-nusB-thiL-pgpA	riboflavin synthase beta chain	TGCATTTAGTGGGTGCA
8	A	443846	41%		2-dehydropantoate reductase, NADPH-specific	panE-yajL	panE	<		>	yajQ	yajQ	predicted nucleotide binding protein	ACCATCAAAGTGTTGAG
9	D	460064	42%		ATPase and specificity subunit of ClpX-ClpP ATP-dependent serine protease		clpX	>	lon	>	hupB	hupB	HU, DNA-binding transcriptional regulator, beta subunit	CTCGGCGAAGTGATGCA
10	B	471846	56%		fused predicted multidrug transporter subunits of ABC superfamily: ATP-binding components		mdlB	>		>	glnK	glnK-amtB	nitrogen assimilation regulatory protein for GlnL, GlnE, and AmtB	TGCACTGTCATAGTGCG
11	D	500472	41%		acetyl esterase	aes	aes	<	gsk	<	ybaL		predicted transporter with NAD(P)-binding Rossmann-fold domain	GGCGGTAATGTCATGCA
12	C		49%		glycerate kinase II		glxK	>		<	ylbA		conserved protein	GGCGTGGAAGTGGTGCA
13	B	547672	44%		predicted acyl-CoA synthetase with NAD(P)-binding Rossmann-fold domain		fdrA	>		>	ylbF	ylbF-ybcF	conserved protein	GGCATTCACATGGTGAC
14	D	635548	47%		conserved protein	ybdM	ybdM	<	ybdN	<	ybdO		predicted DNA-binding transcriptional regulator	GGCGCAATTCTCTTGCA
15	A	655760	41%		anaerobic C4-dicarboxylate transport	dcuC	dcuC	<		>	pagP	pagP	palmitoyl transferase for Lipid A	TGAGTAAATATGTCGCT
16	B	688560	56%		IS5 transposase and trans-activator	insH	insH	<		<	lnt		apolipoprotein N-acyltransferase	TGCACTCTTTTAGTGCT
17	D	719072	47%		putrescine/proton symporter: putrescine/ornithine antiporter	potE	potE	<	speF	>	ybfK	ybfK	hypothetical protein	TGCTACAGGCTGGTGCA
18	D	797452	55%		molybdate transporter subunit		modC	>	ybhA	>	pgl	pgl	6-phosphogluconolactonase	TGTATGATTATCATGCA
19	D	811168	41%		8-amino-7-oxononanoate synthase		bioF	>	bioC	>	bioD	bioD	dethiobiotin synthetase	TTTACCACGCTGGTGCA
20	B	847362	87%		glutamine transporter subunit	glnHPQ	glnH	<		<	dps		Fe-binding and storage protein	TGCCCCAGAATGGTGCA
21	B	894130	95%		putrescine transporter subunit: periplasmic-binding component of ABC superfamily		potF	>		>	potG	potGHI	putrescine transporter subunit: ATP-binding component of ABC superfamily	CGCACCATTATGGTGCG
22	D	933138	43%		DNA-binding transcriptional dual regulator, leucine-binding		lrp	>	ftsK	>	lolA	lolA-rarA	chaperone for lipoproteins	AGCCAGAATATGACGAA
23	D	939430	43%		recombination protein		rarA	>	serS	>	dmsA	dmsABC	dimethyl sulfoxide reductase, anaerobic, subunit A	CTGACTAACCTGGTGCG
24	D	954262	49%		formate transporter	focA-pflB	focA	<	ycaO	>	ycaP	ycaP	conserved inner membrane protein	TGCACGCGTTTGCTGCA
25	D	1004568	54%		predicted periplasmic pilini chaperone		ycbF	>	pyrD	>	zapC	zapC	cell division protein	ATCAATATTATGGCGAA
26	D	1022172	56%		conserved protein		sxy	>	yccS	<	yccF		conserved inner membrane protein	TGCGCCGTTATCGCGCA
27	D	1147452	50%		50S ribosomal subunit protein L32		rpmF	>	plsX	>	fabH	fabHDG-acpP-fabF	3-oxoacyl-[acyl-carrier-protein] synthase III	AGCACAATGCTGGTGCA
28	A	1250156	40%		dihydroxyacetone kinase, N-terminal domain	dhaKLM	dhaK	<		>	dhaR	dhaR	predicted DNA-binding transcriptional regulator, dihydroxyacetone	TGCAATAAGCTGATGAA
29	D	1272070	49%		predicted cation regulator		chaB	>	chaC	<	ychN		conserved protein	AGCTTGATCTTGATGAT
30	B	1308556	42%		voltage-gated potassium channel	kch	kch	<		<	yciI		predicted enzyme	TGCCCTTAAATATTGAA
31	D	1319766	47%		fused glutamine amidotransferase (component II) of anthranilate synthase/anthranilate phosphoribosyl transferase	trpDCBA	trpD	<	trpE	<	trpL		trp operon leader peptide	TGCACGCTTATCGCGCC
32	D	1359448	45%		gamma-Glu-putrescine synthase	puuAP	puuA	<	puuD	>	puuR	puuRCBE	DNA-binding transcriptional repressor	CGCACCTATATGGTGAA
33	D	1402070	92%		predicted peptidase, aminobenzoyl-glutamate utilization protein	abgBT-ogt	abgB	<	abgA	>	abgR	abgR	predicted DNA-binding transcriptional regulator	AACGCCGGAATGATGCA
34	D	1410138	45%		predicted C32 tRNA thiolase	ttcA	ttcA	<	intR	<	ydaQ		Rac prophage; conserved protein	GGCAAAAGTATGCCGCG
35	D	1413348	65%		Rac prophage; recombination and repair protein	recT-ralA-rcbA-xisR-intR	recT	<	recE	<	racC		Rac prophage; predicted protein	TGGATCATGGACGTGAA
36	D	1534050	53%		conserved protein	yddE	yddE	<	narV	<	narW		nitrate reductase 2 (NRZ), delta subunit (assembly subunit)	TTCACCCGACTGGTGCA
37	D	1604568	40%		AI2 transporter		lsrB	>	lsrF	>	lsrG	lsrG-tam	autoinducer-2 (AI-2) modifying protein LsrG	GGCAAAGATATGGTGCG
38	B	1613766	40%		predicted DNA-binding transcriptional regulator		yneJ	>		>	yneK	yneK	predicted protein	TTCAACCTGGTGATGCC
39	A	1630062	44%		predicted mannonate dehydrogenase	ydfI	ydfI	<		>	ydfK	ydfK	Qin prophage; predicted DNA-binding transcriptional regulator	ATCCCCATCATGGTGAT
40	B	1653158	41%		predicted dehydratase	rspAB	rspA	<		<	ynfA		conserved inner membrane protein	TGCGTAGATTTCATGCA
41	D	1683044	100%		sensory histidine kinase in two-component regulatory system with RstA		rstB	>	tus	<	fumC		fumarate hydratase (fumarase C),aerobic Class II	TGCACCTGTATGTTGCA
42	D	1706242	52%		predicted iron-sulfur protein		rsxB	>	rsxC	>	rsxD	rsxDGE-nth	predicted inner membrane oxidoreductase	ATCAGCAATGTTATGCG
43	D	1763330	41%		predicted protein	ydiH	ydiH	<	ydiI	<	ydiJ		predicted FAD-linked oxidoreductase	TTGAACATATTGGTGAT
44	A	1830436	70%		succinylornithine transaminase, PLP-dependent	astCADBE	astC	<		>	xthA	xthA	exonuclease III	TGCGCCATTCTGACGCA
45	D	1853134	40%		predicted DNA-binding transcriptional regulator	ydjF	ydjF	<	ydjG	<	ydjH		predicted kinase	GGCAGCCACTTTGTGCT
46	D	1861262	65%		methionine sulfoxide reductase B	msrB	msrB	<	gapA	>	yeaD	yeaD	conserved protein	TTCACGCTACTACCGCT
47	B	1863654	50%		predicted oxidoreductase	yeaE	yeaE	<		<	mipA		scaffolding protein for murein synthesizing machinery	TGCATTACGGTGGTGCA
48	D	1864146	49%		methylglyoxal reductase	yeaE	yeaE	<	mipA	>	yeaG	yeaGH	conserved protein with nucleoside triphosphate hydrolase domain	GTCATCCAGGTGACGCA
49	C	1876962	51%		predicted diguanylate cyclase		yeaP	>		<	yeaQ		conserved inner membrane protein	CTCAGGAATGTAGCGCT
50	D	1951952	40%		predicted methyltransferase		cmoA	>	cmoB	<	torZ		trimethylamine N-oxide reductase system III, catalytic subunit	ATCAGAAAGATGGGGCA
51	A	2060070	50%		DNA-binding transcriptional dual regulator of nitrogen assimilation	nac	nac	<		>	asnV	asnV	Asn tRNA	TGAACCATCGTGGTGCA
52	D	2122744	47%		mannose-1-phosphate guanyltransferase	cspB	cpsB	<	wcaI	<	gmm		GDP-mannose mannosyl hydrolase	GGGATAATTATGGCGCA
53	B	2184766	45%		membrane protein conferring nickel and cobalt resistance		rcnA	>		>	rcnB	rcnB	periplasmic protein involved in nickel/cobalt export	TGCTCGCTAAGGATGCT
54	D	2212172	70%		predicted response regulator in two-component system withYehU	yehT	yehT	<	yehU	>	mlrA	mlrA	DNA-binding transcriptional regulator	GTCGCTGAAATGGTGCA
55	D	2214150	46%		predicted protein		yohO	>	yehW	<	yehX		predicted transporter subunit: ATP-binding component of ABC superfamily	TGCAAAACTTTCTTGCT
56	A	2458968	47%		conserved protein	yfcZ	yfcZ	<		>	fadL	fadL	long-chain fatty acid outer membrane transporter	TTGTCCATAATGGTGCC
57	D	2489132	40%		predicted transporter	yfdVE	yfdV	<	oxc	<	frc		formyl-CoA transferase, NAD(P)-binding	TTCGTGAATTTATTGAA
58	D	2504132	51%		predicted peptidase	ypdE-fryA	ypdE	<	ypdF	<	fryC		predicted enzyme IIC component of PTS	TGCGCCCTGCTGGCGCA
59	D	2511644	64%		manganese/divalent cation transporter	mntH	mntH	<	nupC	>	insL	insL	IS186/IS421 transposase	AGCACCTTTATCGTGCT
60	A	2529354	78%		cell division protein involved in Z ring assembly	zipA	zipA	<		>	cysZ	cysZ	predicted inner membrane protein	TTCGTTATTTTACCGCT
61	D	2610536	41%		predicted processing element hydrogenase 4		hyfJ	>	hyfR	>	focB	focB	predicted formate transporter	CGCAGCCGTTAGATGCA
62	D	2664968	54%		stationary phase inducible protein		csiE	>	hcaT	<	hcaR		DNA-binding transcriptional activator of 3-phenylpropionic acid catabolism	TGCGTTATATTGCTGCT
63	D	2678844	42%		predicted sugar transporter subunit: periplasmic-binding component of ABC superfamily	yphFED	yphF	<	yphG	>	yphH	yphH	predicted DNA-binding transcriptional regulator	CGCGAGAAACTGCTGCG
64	D	2905372	41%		predicted protein		ygcG	>	eno	<	pyrG		CTP synthetase	TTCAGCCGGTTGGCGCG
65	D	2912460	64%		(p)ppGpp synthetase I/GTP pyrophosphokinase	relA-mazEFG	relA	<	rumA	>	barA	barA	hybrid sensory histidine kinase, in two-component regulatory system with UvrY	ATGACCAAGATGGCGCA
66	B	3001538	44%		xanthine dehydrogenase, FAD-binding subunit		xdhB	>		>	xdhC	xdhC	xanthine dehydrogenase, Fe-S binding subunit	ATCCCGTTAATGGTGCA
67	D	3002666	45%		xanthine dehydrogenase, Fe-S binding subunit		xdhC	>	ygeV	>	ygeW	ygeW	conserved protein	TGCCTCAATAGCGCGCA
68	D	3118836	51%		predicted inner membrane lipoprotein	yghJ	yghJ	<	glcA	<	glcB		malate synthase G	CGTTCCACATTGGCGCA
69	D	3178240	51%		transport channel		tolC	>	ygiB	>	ygiC	ygiC	predicted enzyme	TTGCTGAATTTGGTGAA
70	D	3179938	53%		predicted enzyme		ygiC	>	ygiD	>	zupT	zupT	zinc transporter	AGCGGTAACGTGGTGCA
71	D	3262568	46%		propionate kinase/acetate kinase C, anaerobic	tdcDEFG	tdcD	<	tdcC	<	tdcB		catabolic threonine dehydratase, PLP-dependent	AGCACCAGGCTGGCGAT
72	D	3391344	41%		predicted peptidase	tldD	tldD	<	yhdP	<	rng		ribonuclease G	CGCCCAAATACGATGCA
73	B	3446170	40%		50S ribosomal subunit protein L14	rplNXE-rpsNH-rplFR-rpsE-rpmD-rplO-secY-rpmJ	rplN	<		<	rpsQ		30S ribosomal subunit protein S17	TGGATCATTTTAGTGCT
74	D	3565536	64%		glycogen phosphorylase	glgP	glgP	<	glgA	<	glgC		glucose-1-phosphate adenylyltransferase	TGCATGCCAGTCGTGCG
75	D	3629462	47%		predicted HlyD family secretion protein	yhiI-rbbA-yhhJ	yhiI	<	yhiJ	>	yhiM	yhiM	conserved inner membrane protein	TAAATCAAGTTGTTGCA
76	D	3633862	50%		predicted protein	yhiJ	yhiJ	<	yhiM	<	yhiN		predicted oxidoreductase with FAD/NAD(P)-binding domain	GGCCAGATCTTTATGAA
77	D	3659166	43%		multidrug resistance efflux transporter		mdtE	>	mdtF	<	gadW		DNA-binding transcriptional activator	CGAACCATCTTGCTGAA
78	D	3688672	45%		endo-1,4-D-glucanase	bcsZC	bcsZ	<	bcsB	<	bcsA		cellulose synthase, catalytic subunit	ATCGACCTATTGGTGCA
79	D	3713740	58%		predicted acyltransferase with acyl-CoA N-acyltransferase domain		yiaC	>	bisC	>	yiaD	yiaD	predicted outer membrane lipoprotien	GGCACCGATGTTGCGCT
80	D	3753568	43%		conserved inner membrane protein	yiaWV	yiaW	<	aldB	<	yiaY		predicted Fe-containing alcohol dehydrogenase	CTCGACAGCGTGACGCA

81	D	3805372	48%		glucosyltransferase I	*rfaG*	*rfaG*	<	*rfaQ*	>	*waaA*	*waaA*	3-deoxy-D-manno-octulosonic-acid transferase (KDO transferase)	TTAGTCTATTTGGTGCA
82	D	3878632	42%		DNA gyrase, subunit B	*gyrB*	*gyrB*	<	*recF*	<	*dnaN*		DNA polymerase III, beta subunit	TGGAACGTAATTTTGAA
83	B	3933336	59%		fused D-ribose transporter subunits of ABC superfamily: ATP-binding components		*rbsA*	>		>	*rbsC*	*rbsCBKR*	D-ribose transporter subunit	TGCATGACGATGATGCG
84	B	3994336	40%		conserved protein		*yigA*	>		>	*xerC*	*xerC-yigB*	site-specific tyrosine recombinase	TACAGCAAAATTGCGCA
85	A	4056244	97%		glutamine synthetase	*glnALG*	*glnA*	<		>	*typA*	*typA*	GTP-binding protein	AGCACCATGTTGGTGCA
86	D	4138946	40%		fructose-6-phosphate aldolase 2	*fsaB-gldA*	*fsaB*	<	*ptsA*	>	*frwC*	*frwCB*	predicted enzyme IIC component of PTS	CGCACAATAATGCTGCG
87	D	4140848	60%		fused predicted PTS enzymes: Hpr component/enzyme I component/enzyme IIA component	*ptsA-fsaB-gldA*	*ptsA*	<	*frwC*	>	*frwB*	*frwB*	predicted enzyme IIB component of PTS	GGCGGCATCGTGGTGCA
88	D	4157268	60%		ncRNA	*oxyS*	*oxyS*	<	*oxyR*	<	*sthA*		pyridine nucleotide transhydrogenase, soluble	GGGATCACTTTACTGCC
89	A	4173336	63%		pantothenate kinase	*coaA*	*coaA*	<		>	*thrU*	*thrU-tyrU-glyT-thrT-tufB*	Thr tRNA	AGCACAATGATGTTGAA
90	D	4201970	51%		sensory histidine kinase in two-component regulatory system with ZraR		*zraS*	>	*zraR*	<	*purD*		phosphoribosylglycinamide synthetase phosphoribosylamine-glycine ligase	ATCGCGATCTTGCCGCA
91	B	4260864	58%		tRNA-dihydrouridine synthase A		*dusA*	>		>	*pspG*	*pspG*	phage shock protein G	GGCGTTATTTAAGTGAA
92	B	4304842	43%		predicted alkyl sulfatase	*yjcS*	*yjcS*	<		<	*alsK*		D-allose kinase	TGCGCTTTATTGCCGCC
93	B	4530150	40%		KpLE2 phage-like element; predicted endoglucanase with Zn-dependent exopeptidase domain	*sgcXBCQAER*	*sgcX*	<		<	*yjhP*		KpLE2 phage-like element; predicted methyltransferase	TGCAACAAAGTGGTGCT
	A 11	Cut-off	> 40%				Spacer = 32							
	B 19	Total	93				ORF = 60							
	C 2													
	D 60													

Table S4

RpoN holoenzyme-NtrC mixture-binding sites on the *E. coli* K-12 genome

No	gSELEX peak type	Map position (bp)	RpoN holo + NtrC	Group	Regulon RpoN	Regulon NtrC	ChIP-chip	Left Gene Function	Operon	Left Gene	D	RpoN + NtrC	D	RightGene	Operon	Right Gene Function	RpoN promoter motif (ntGGcacnnnnnttGCn)	NtrC box (TGCACCAnnnTGGTGCA)
1	A	42372	30%					predicted transporter	*caiTABCDE*	*caiT*	<		>	*fixA*	*fixABCX*	predicted electron transfer flavoprotein subunit, ETFP adenine nucleotide-binding domain	TTGGCATCGGCTTTGCT	
2	A	77346	43%	II-A				DNA-binding transcriptional regulator	*sgrR-sroA-tbpA-thiPO*	*sgrR*	<		>	*sgrS*	*sgrST-setA*	ncRNA	CTGGCAGCGTTATTGAG	
3	D	91366	42%					S-adenosyl-dependent methyltransferase activity on membrane-located substrates		*mraW*	>	*ftsL*	>	*ftsI*	*ftsI-murEF-mraY-murD-ftsW-murGC-ddlB-ftsQAZ-lpxC*	transpeptidase involved in septal peptidoglycan synthesis (penicillin-binding protein 3)	CTGGCGCTGGCTTTTCT	
4	D	159632	30%					poly(A) polymerase I	*pcnB-folK*	*pcnB*	<	*yadB*	<	*dksA*		DNA-binding transcriptional regulator of rRNA transcription, DnaK suppressor protein	TCGGCGTGAATAATGCC	
5	B	257850	30%					hydrolase, binds to enzyme IIA(Glc)		*frsA*	>		>	*crl*	*crl*	DNA-binding transcriptional regulator	TTGGTAAAACAGTTGCA	
6	D	261272	43%					gamma-glutamate kinase		*proB*	>	*proA*	>	*thrW*	*thrW*	Thr tRNA	ACGGCACAGTTTATGCA	
7	A	347864	43%					DNA-binding transcriptional activator	*prpR*	*prpR*	<		>	*prpB*	*prpBCDE*	2-methylisocitrate lyase	GTGGCACACCCCTTGCT	
8	D	415552	55%					exonuclease, dsDNA, ATP-dependent	*sbcC*	*sbcC*	<	*sbcD*	>	*phoB*	*phoBR*	DNA-binding response regulator in two-component regulatory system with PhoR (or CreC)	CCGGCACAAAACTTTCC	
9	B	418832	37%					sensory histidine kinase in two-component regulatory system with PhoB		*phoR*	>		>	*brnQ*	*brnQ-proY*	predicted branched chain amino acid transporter (LIV-II)	TTGGCACAAAAATTTAA	
10	D	437072	34%					phosphatidylglycerophosphatase A		*pgpA*	>	*yajO*	<	*dxs*		1-deoxyxylulose-5-phosphate synthase, thiamine-requiring, FAD-requiring	TCGGCATGGATTATGTC	
11	B	471846	67%					fused predicted multidrug transporter subunits of ABC superfamily: ATP-binding components		*mdlB*	>		>	*glnK*	*glnK-amtB*	nitrogen assimilation regulatory protein for GlnL, GlnE, and AmtB	CTGGCACACCGCTTGCA	TGCACTGTCATAGTGCG
12	D	503730	64%					predicted transporter with NAD(P)-binding Rossmann-fold domain	*ybaL*	*ybaL*	<	*fsr*	>	*ushA*	*ushA*	bifunctional UDP-sugar hydrolase/5'-nucleotidase	ATGCCATGGTCGTTGCC	
13	D	601842	33%					copper/silver efflux system, membrane component		*cusA*	>	*pheP*	<	*ybdG*		predicted mechanosensitive channel	TTGGAATGCTTTTTTCC	
14	B	619432	34%					iron-enterobactin transporter subunit	*fepC*	*fepC*	<		<	*fepG*		iron-enterobactin transporter subunit	TTGGCCCGATAATTGCC	
15	A	655760	50%					anaerobic C4-dicarboxylate transport	*dcuC*	*dcuC*	<		>	*pagP*	*pagP*	palmitoyl transferase for Lipid A	TTGGTAAAGTTTTTGCT	TGAGTAAATATGTCGCT
16	B	688560	67%					IS5 transposase and trans-activator	*insH*	*insH*	<		<	*lnt*		apolipoprotein N-acyltransferase	TTGGCACATCTATTGCT	TGCACTCTTTTAGTGCT
17	D	700256	39%	II-D				UMP phosphatase	*nagD*	*nagD*	<	*nagC*	<	*nagA*		N-acetylglucosamine-6-phosphate deacetylase	ATGTAATGAATTTTGCC	
18	D	702662	53%					N-acetylglucosamine-6-phosphate deacetylase	*nagAC-umpH*	*nagA*	<	*nagB*	>	*nagE*	*nagE*	fused N-acetyl glucosamine specific PTS enzyme: IIC, IIB , and IIA components	CTGGCCTGCTTTATGCA	
19	A	784656	39%					conserved protein	*ybgS*	*ybgS*	<		>	*aroG*	*aroG*	3-deoxy-D-arabino-heptulosonate-7-phosphate synthase, phenylalanine repressible	CTGGCGTTTTTCTTGCT	
20	B	847362	78%					glutamine transporter subunit	*glnHPQ*	*glnH*	<		<	*dps*		Fe-binding and storage protein	CTGGCACGATTTTTTCA	TGCCCCAGAATGGTGCA
21	D	848670	55%					Fe-binding and storage protein	*dps*	*dps*	<	*rhtA*	>	*ompX*	*ompX*	outer membrane protein	TTGGCACGAAAATTAAC	
22	A	882830	72%					undecaprenyl pyrophosphate phosphatase	*ybjG*	*ybjG*	<		>	*cmr*	*cmr*	multidrug efflux system protein	TTGGCGAAGAAATTGCA	
23	D	884934	36%					predicted protein	*ybjH*	*ybjH*	<	*ybjI*	<	*ybjJ*		predicted transporter	TAGGCACTATTTTTTCC	
24	B	891170	32%					nitroreductase A, NADPH-dependent, FMN-dependent		*nfsA*	>		>	*rimK*	*rimK-ybjN*	ribosomal protein S6 modification protein	TTGGCACCGCCATTACC	
25	B	892632	74%					predicted oxidoreductase		*ybjN*	>		>	*potF*	*potFGHI*	putrescine transporter subunit: periplasmic-binding component of ABC superfamily	TTTGCATGGTTCGTGCG	
26	B	894130	45%	I-B				putrescine transporter subunit: periplasmic-binding component of ABC superfamily		*potF*	>		>	*potG*	*potGHI*	putrescine transporter subunit: ATP-binding component of ABC superfamily	ATGGTCGGAAATATGCG	CGCACCATTATGGTGCG
27	D	1022172	39%	I-D				conserved protein		*sxy*	>	*yccS*	<	*yccF*		conserved inner membrane protein	ATGGCGCGTAAATTGTT	TGCGCCGTTATCGCGCA
28	A	1073268	73%					predicted monooxygenase	*rutABCDEFG*	*rutA*	<		>	*rutR*	*rutR*	predicted DNA-binding transcriptional regulator	CTGGCATCCGCTTTGCA	
29	D	1087762	61%					predicted glycosyl transferase	*pgaCD*	*pgaC*	<	*pgaB*	<	*pgaA*		predicted outer membrane protein	TTGGCATGTTATATGAA	
30	D	1155742	30%					thymidylate kinase		*tmk*	>	*holB*	>	*ycfH*	*ycfH*	predicted metallodependent hydrolase	ATGGCACGCTATATGCC	
31	B	1177842	30%					outer membrane-specific lipoprotein transporter subunit		*lolE*	>		>	*nagK*	*nagK-cobB*	N-acetyl-D-glucosamine kinase	ATGGTACGCTGTATGCC	
32	D	1213734	32%					DNA-binding transcriptional regulator		*bluR*	<	*ycgF*	>	*ycgZ*	*ycgZ-ymgA-ariR-ymgC*	predicted protein	TTGGCGCAGGTTTTGCT	
33	B	1308556	54%					voltage-gated potassium channel	*kch*	*kch*	<		<	*yciI*		predicted enzyme	AGGGCACGGTTTTTGCA	TGCCCTTAAATATTGAA
34	D	1311268	31%					predicted inner membrane protein	*yciB*	*yciB*	<	*yciC*	>	*ompW*	*ompW*	outer membrane protein W	CTGGCACCGGTGATGTT	
35	A	1366070	77%					DNA-binding transcriptional activator	*pspF*	*pspF*	<		>	*pspA*	*pspABCDE*	regulatory protein for phage-shock-protein operon	TTGGCACGCAAATTGTA	
36	D	1462866	42%					DNA-binding transcriptional repressor of phenylacetic acid degradation, aryl-CoA responsive		*paaX*	>	*paaY*	<	*insD*		KpLE2 phage-like element; IS2 insertion element transposase InsAB'	TTGGCATGGAAAAAGCA	
37	D	1493450	36%					predicted DNA-binding transcriptional regulator	*ydcI*	*ydcI*	<	*ydcJ*	>	*mdoD*	*mdoD*	glucan biosynthesis protein, periplasmic	CGGGAACAGTTTTCGCA	
38	B	1561132	44%	II-B				D-ala-D-ala dipeptidase, Zn-dependent	*ddpXABCDF*	*ddpX*	<		<	*dos*		cAMP phosphodiesterase, heme-regulated	ATGGCATGAGATCTGCA	
39	D	1576570	32%					predicted porin protein	*yddB*	*yddB*	<	*yddA*	<	*ydeM*		conserved protein	ATGGTATGAAAATTGTC	
40	B	1608732	38%					altronate oxidoreductase, NAD-dependent	*uxaB*	*uxaB*	<		<	*yneF*		predicted diguanylate cyclase	CTGGCGTGGTCGTTGTT	
41	B	1678972	32%					predicted arginine/ornithine antiporter transporter		*ydgI*	>		>	*folM*	*folM*	dihydrofolate reductase isozyme	ATGGACTGATTAATGCA	
42	D	1683044	36%	I-D				sensory histidine kinase in two-component regulatory system with RstA		*rstB*	>	*tus*	<	*fumC*		fumarate hydratase (fumarase C),aerobic Class II	TCGGCATCGTAATTTAA	TGCACCTGTATGTTGCA
43	B	1709534	43%	II-B				predicted inner membrane NADH-quinone reductase		*rsxE*	>		>	*nth*	*nth*	DNA glycosylase and apyrimidinic (AP) lyase (endonuclease III)	ATGGCACATTGTTTGAC	
44	D	1790536	49%					conserved protein	*ydiV*	*ydiV*	<	*nlpC*	<	*btuD*		vitamin B12 transporter subunit : ATP-binding component of ABC superfamily	TTGGCACGCAAATTGAT	
45	A	1830436	54%					succinylornithine transaminase, PLP-dependent	*astCADBE*	*astC*	<		>	*xthA*	*xthA*	exonuclease III	ATGGCGCAGTAATTTCC	TGCGCCATTCTGACGCA
46	D	1838132	32%					predicted transporter subunit: ATP-binding component of ABC superfamily		*ynjD*	>	*ynjE*	<	*ynjF*		predicted phosphatidyl transferase, inner membrane protein	GTGGCGCAGATTATGCT	
47	B	1863654	50%	I-B				methylglyoxal reductase	*yeaE*	*yeaE*	<		<	*mipA*		scaffolding protein for murein synthesizing machinery	CTGGCCTGATTTCTACC	TGCATTACGGTGGTGCA
48	D	1869670	85%					conserved protein		*yeaH*	>	*yeaI*	>	*yeaJ*	*yeaJ*	predicted diguanylate cyclase	TTGGCACGGTTTTTTCA	
49	B	1905652	39%	II-B				predicted protein	*yobF-cspC*	*yobF*	<		<	*yebO*		predicted protein	ATGGCAAGCCATTTTTA	
50	D	1982556	32%					fused L-arabinose transporter subunits of ABC superfamily: membrane components	*araH*	*araH*	<	*araG*	<	*araF*		L-arabinose transporter subunit	TTGGTATGGATATTGAC	
51	D	2034858	30%					Hsp31 molecular chaperone		*hchA*	>	*yedV*	<	*yedW*		predicted DNA-binding response regulator in two-component system with YedV	CCGGAACACATTATGCT	
52	A	2036832	33%					predicted DNA-binding response regulator in two-component system with YedV	*yedWV*	*yedW*	<		>	*hiuH*	*hiuH*	hydroxyisourate hydrolase / transthyretin-related protein	ATGGCATATTATTTTCA	
53	A	2060070	86%					DNA-binding transcriptional dual regulator of nitrogen assimilation	*nac*	*nac*	<		>	*asnV*	*asnV*	Asn tRNA	CTGGCAAGCATCTTGCA	TGAACCATCGTGGTGCA
54	D	2212172	33%	I-D				predicted response regulator in two-component system withYehU	*yehT*	*yehT*	<	*yehU*	>	*mlrA*	*mlrA*	DNA-binding transcriptional regulator	GCGGCGCTGTTTATGCG	GTCGCTGAAATGGTGCA
55	D	2317672	34%					DNA-binding response regulator in two-component regulatory system with RcsC and YojN		*rcsB*	>	*rcsC*	>	*atoS*	*atoSC*	sensory histidine kinase in two-component regulatory system with AtoC	TTGGCGTGGTTTATGCG	
56	D	2318844	52%					hybrid sensory kinase in two-component regulatory system with RcsB and YojN	*rcsC*	*rcsC*	<	*atoS*	>	*atoC*	*atoC*	fused response regulator of ato operon, in two-component system with AtoS: response regulator/sigma54 interaction protein	CTGGCACAAAATATTCC	
57	B	2321470	77%					fused response regulator of ato operon, in two-component system with AtoS: response regulator/sigma54 interaction protein		*atoC*	>		>	*atoD*	*atoDAEB*	acetyl-CoA:acetoacetyl-CoA transferase, alpha subunit	CTGGCACTCCCCTTGCT	
58	B	2360468	34%					predicted DNA-binding transcriptional regulator	*yfaX-rhmD-yfaVU*	*yfaX*	<		<	*yfaY*		conserved protein	ATGGCACTGAATATGCT	
59	D	2366462	32%					undecaprenyl phosphate-L-Ara4FN transferase		*arnC*	>	*arnA*	>	*arnD*	*arnDTEF*	conserved protein	ATGGCACAATTTATGAT	
60	B	2425832	55%					lysine/arginine/ornithine transporter subunit	*argT-hisJQMP*	*argT*	<		<	*ubiX*		3-octaprenyl-4-hydroxybenzoate carboxy-lyase	ATGGCATAAGACCTGCA	
61	D	2427232	34%					3-octaprenyl-4-hydroxybenzoate carboxy-lyase	*ubiX*	*ubiX*	<	*purF*	<	*cvpA*		membrane protein required for colicin V production	TGGGCGAGAAAATTGCC	
62	B	2429072	32%					membrane protein required for colicin V production	*cvpA-purF-ubiX*	*cvpA*	<		<	*dedD*		conserved protein	TCGGTGCAGCTTTTTCT	
63	A	2458968	33%	I-A				conserved protein	*yfcZ*	*yfcZ*	<		>	*fadL*	*fadL*	long-chain fatty acid outer membrane transporter	CTGGAGCAGCACTTTCC	TTGTCCATAATGGTGCC
64	A	2493362	46%					predicted inner membrane protein	*yfdY*	*yfdY*	<		>	*lpxP*	*lpxP*	palmitoleoyl-acyl carrier protein (ACP)-dependent acyltransferase	ACAGCATGTTAAATGCA	
65	D	2511644	40%	I-D				manganese/divalent cation transporter	*mntH*	*mntH*	<	*nupC*	>	*insL*	*insL*	IS186/IS421 transposase	CTGGTACTGAACATGTT	AGCACCTTTATCGTGCT
66	B	2520564	59%					DNA-binding transcriptional activator	*xapR*	*xapR*	<		<	*xapB*		xanthosine transporter	ATGCCATGATAATTTAA	
67	B	2522072	36%					xanthosine transporter	*xapB*	*xapB*	<		<	*xapA*		purine nucleoside phosphorylase II	TTGGTGGGGATGTTGTT	
68	B	2531464	48%					cysteine synthase A, O-acetylserine sulfhydrolase A subunit		*cysK*	>		>	*ptsH*	*ptsHI-crr*	phosphohistidinoprotein-hexose phosphotransferase component of PTS system (Hpr)	CTGGCATTACTGTTGCA	
69	D	2584448	33%					fused predicted oxidoreductase: FeS binding subunit/NAD/FAD-binding subunit	*aegA*	*aegA*	<	*narQ*	>	*acrD*	*acrD*	aminoglycoside/multidrug efflux system	ACGGTACAATTTATGCA	
70	B	2599140	100%					thiol peroxidase, thioredoxin-dependent		*bcp*	>		>	*hyfA*	*hyfABCDEFGHIJR-focB*	hydrogenase 4, 4Fe-4S subunit	ATGGCATCCTTTATGCA	
71	D	2608134	30%					hydrogenase 4, membrane subunit		*hyfF*	>	*hyfG*	>	*hyfH*	*hyfHIJR-focB*	hydrogenase 4, Fe-S subunit	ACGGCACGGTTTTTGAC	
72	B	2689364	47%					ncRNA	*glmY*	*glmY*	<		<	*purL*		phosphoribosylformyl-glycineamide synthetase	TTGGCACAGTTACTGCA	
73	D	2733670	43%					conserved protein	*yfiH*	*yfiH*	<	*rluD*	>	*bamD*	*bamD*	predicted lipoprotein	CCGGAACGGTTTTTGCC	
74	B	2825748	40%					glucitol/sorbitol-specific enzyme IIA component of PTS		*srlB*	>		>	*srlD*	*srlD-gutM-srlR-gutQ*	sorbitol-6-phosphate dehydrogenase	GTGCCACATTTGCGGCT	
75	A	2830336	30%					DNA-binding transcriptional activator	*norR*	*norR*	<		>	*norV*	*norVW*	flavorubredoxin oxidoreductase	CTGGCACGCAATCTGCA	
76	B	2836270	36%					formate dehydrogenase-H, [4Fe-4S] ferredoxin subunit	*hydN-hypF*	*hydN*	<		<	*ascG*		DNA-binding transcriptional repressor	CTGGCATGATTTGTGAA	
77	D	2846068	31%					hydrogenase 3, membrane subunit	*hycDEFGHI*	*hycD*	<	*hycC*	<	*hycB*		hydrogenase 3, Fe-S subunit	GTGGCGCGTTTGTTGCC	
78	A	2848650	38%					regulator of the transcriptional regulator FhlA	*hycABCDEFGHI*	*hycA*	<		>	*hypA*	*hypABCDE-fhlA*	protein involved in nickel insertion into hydrogenases 3	CTGGCACAATTATTGCT	
79	D	2912460	36%	I-D				(p)ppGpp synthetase I/GTP pyrophosphokinase	*relA-mazEFG*	*relA*	<	*rumA*	>	*barA*	*barA*	hybrid sensory histidine kinase, in two-component regulatory system with UvrY	CTGGAACGCTTTTCGCA	ATGACCAAGATGGCGCA
80	D	2950142	31%					fused acetylglutamate kinase homolog (inactive)/amino acid N-acetyltransferase		*argA*	>	*recD*	<	*recB*		exonuclease V (RecBCD complex), beta subunit	CTGGCAGCGTTAATTCA	
81	A	3004270	55%					predicted DNA-binding transcriptional regulator	*ygeV*	*ygeV*	<		>	*ygeW*	*ygeW*	conserved protein	CTGGCACACTTATTGTT	
82	D	3012642	31%					conserved protein with NAD(P)-binding Rossman fold	*yqeB*	*yqeB*	<	*yqeC*	>	*mocA*	*mocA*	molybdenum cofactor cytidylyltransferase	CTGGCCCGTGGTTTTCT	
83	B	3043930	33%					predicted NAD(P)-binding oxidoreductase with NAD(P)-binding Rossmann-fold domain	*ygfF*	*ygfF*	<		<	*gcvP*		glycine decarboxylase, PLP-dependent, subunit (protein P) of glycine cleavage complex	CTGGCGCGAATATTTAC	
84	D	3262568	35%	I-D				propionate kinase/acetate kinase C, anaerobic	*tdcDEFG*	*tdcD*	<	*tdcC*	<	*tdcB*		catabolic threonine dehydratase, PLP-dependent	ATGGCGTTACTATTACC	AGCACCAGGCTGGCGAT
85	B	3370654	46%					sialic acid transporter	*nanTEK-yhcH*	*nanT*	<		<	*nanA*		N-acetylneuraminate lyase	CTGGCAGATATTTTTCA	
86	B	3408032	33%					methylase for 50S ribosomal subunit protein L11		*prmA*	>		>	*dusB*	*dusB-fis*	tRNA-dihydrouridine synthase B	TTGGCACGCAAATTTGC	
87	B	3417032	43%					predicted outer membrane protein		*yhdV*	>		>	*yhdX*	*yhdXYZ*	predicted amino-acid transporter subunit	CTGGCACTACTTTTGCT	
88	B	3440634	32%					30S ribosomal subunit protein S13	*rpsMKD-rpoA-rplQ*	*rpsM*	<		<	*rpmJ*		50S ribosomal subunit protein L36	TTGGCGCTGTTTATGCT	
89	D	3565536	55%					glycogen phosphorylase	*glgP*	*glgP*	<	*glgA*	<	*glgC*		glucose-1-phosphate adenylyltransferase	AAGGCATGTTTTATGCA	TGCATGCCAGTCGTGCG
90	B	3598870	53%					RNA polymerase, sigma 32 (sigma H) factor	*rpoH*	*rpoH*	<		<	*ftsX*		predicted transporter subunit: membrane component of ABC superfamily	CTGGCACAGTTGTTGCT	
91	D	3803540	46%					lipopolysaccharide core biosynthesis protein	*rfaS*	*rfaS*	<	*rfaP*	<	*rfaG*		glucosyltransferase I	ATGGTGCGTAAAATGCA	
92	B	3809172	30%	II-B				formamidopyrimidine/5-formyluracil/ 5-hydroxymethyluracil DNA glycosylase	*mutM*	*mutM*	<		<	*rpmG*		50S ribosomal subunit protein L33	CTGGATCGAATTTTTTC	
93	A	3851352	65%					ncRNA	*istR*	*istR*	<		>	*tisB*	*tisB*	lexA-regulated toxic peptide	TCGGCACGAATTTTGAC	
94	D	3921366	30%					ATP synthase, membrane-bound accesory subunit		*atpI*	<	*gidB*	<	*mnmG*		glucose-inhibited cell-division protein	GTGGCACCGTATCTGCA	
95	D	3937930	40%					DNA-binding transcriptional repressor of ribose metabolism		*rbsR*	>	*hsrA*	<	*yieP*		predicted transcriptional regulator	TTGGCGTGCGTCGTGCA	

96	B	3967058	51%					UDP-GlcNAc:undecaprenylphosphate GlcNAc-1-phosphate transferase		*rfe*	>		>	*wzzE*	*wzzE-wecBC-rffGHCA-wzxE-rffT-wzvE-rffM*	Entobacterial Common Antigen (ECA) polysaccharide chain length modulation protein	CAGGCACGCTTAATGCA	
97	B	4008248	42%					lysophospholipase L(2)		*pldB*	>		>	*yigL*	*yigL*	predicted hydrolase	ATGGCACGTTACTTTCT	
98	A	4056244	84%					glutamine synthetase	*glnALG*	*glnA*	<		>	*typA*	*typA*	GTP-binding protein	TTGGCACAGATTTCGCT	AGCACCATGTTGGTGCA
99	D	4062664	30%					predicted outer membrane porin L	*ompL*	*ompL*	<	*yihO*	<	*yihP*		predicted transporter	TTGGCACGGCCTTTGTA	
100	B	4131538	33%					5,10-methylenetetrahydrofolate reductase		*metF*	>		>	*katG*	*katG*	catalase/hydroperoxidase HPI(I)	TTGGCGCGAATATTGCC	
101	A	4199860	64%					Zn-binding periplasmic protein	*zraP*	*zraP*	<		>	*zraS*	*zraSR*	sensory histidine kinase in two-component regulatory system with ZraR	ATGGCATGATTTCTGCT	
102	B	4260864	50%					tRNA-dihydrouridine synthase A		*dusA*	>		>	*pspG*	*pspG*	phage shock protein G	TCGGCACTTTTGGTGCT	GGCGTTATTTAAGTGAA
103	B	4297530	59%					formate dehydrogenase-H, selenopolypeptide subunit	*fdhF*	*fdhF*	<		<	*mdtP*		predicted outer membrane factor of efflux pump	GTGGCATAAAAGATGCA	
104	B	4304842	41%	I-B				predicted alkyl sulfatase	*yjcS*	*yjcS*	<		<	*alsK*		D-allose kinase	ACGGCGCAGGTACTTTT	TGCGCTTTATTGCCGCC
105	B	4331330	55%					sensory histidine kinase in two-component regulatory system with BasR	*basS*	*basS*	<		<	*basR*		DNA-binding response regulator in two-component regulatory system with BasS	TCGGCGCAGAAAATGCA	
106	D	4409770	36%					predicted transcriptional regulator effector protein		*yjfJ*	>	*yjfK*	>	*yjfL*	*yjfLMC*	conserved inner membrane protein	CTGGCAGCGATTTTTTA	
107	D	4501260	34%					KpLE2 phage-like element; predicted protein		*yjgZ*	>	*insG*	>	*nanX*	*nanXY*	KpLE2 phage-like element; putative sialic acid transporter	TTGGCATGGCGCTTGAG	
108	D	4549850	36%					fructuronate transporter	*gntP*	*gntP*	<	*uxuA*	>	*uxuB*	*uxuB*	D-mannonate oxidoreductase, NAD-binding	TTGGCACGCTTTCTACG	
	A 19		> 30%							Spacer, 61								
	B 42		108							ORF, 47								
	C 0																	
	D 47																	
